# Supplementary material for: Haloperidol bound D2 dopamine receptor structure inspired the discovery of subtype selective ligands
Source: Nat Commun. 2020 Feb 26;11:1074. doi: 10.1038/s41467-020-14884-y (PMC7044277; doi:10.1038/s41467-020-14884-y)
Supplement: Supplementary file 1 — Supplementary Information [file 41467_2020_14884_MOESM1_ESM.pdf]

Supplementary Information

Haloperidol bound D_2 dopamine receptor structure inspired the discovery of subtype selective ligands

Luyu Fan[1], Liang Tan[2], Zhangcheng Chen[1], Jianzhong Qi[1], Fen Nie[1], Zhipu Luo[3], Jianjun Cheng[2] and Sheng Wang[1]

[1]State Key Laboratory of Molecular Biology, CAS Center for Excellence in Molecular Cell Science, Shanghai Institute of Biochemistry and Cell Biology, Chinese Academy of Sciences; University of Chinese Academy of Sciences, 320 Yueyang Road, Shanghai 200031, China

[2]iHuman Institute, ShanghaiTech University, 393 Middle Huaxia Road, Shanghai 201210, China

[3]Institute of Molecular Enzymology, Soochow University, Suzhou, Jiangsu 215123, China

These authors contributed equally: Luyu Fan, Liang Tan

Correspondence and requests for materials should be addressed to J.C. (email: chengjj@shanghaitech.edu.cn) or to S.W. (email: wangsheng@sibcb.ac.cn)

Supplementary Figures

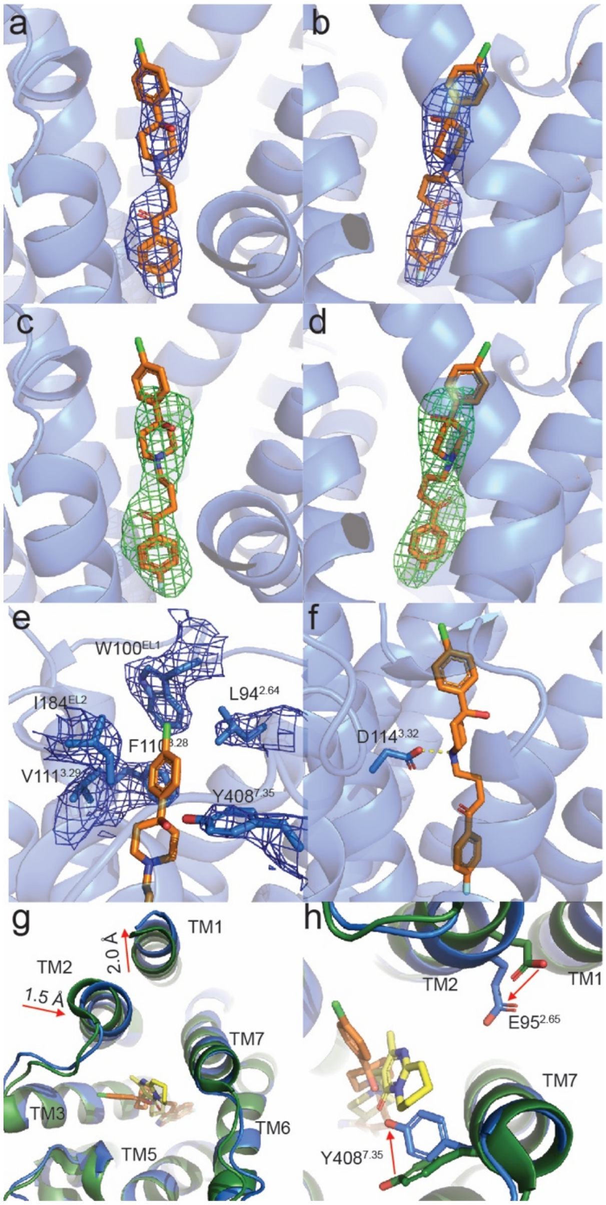

Supplementary Figure 1. Representative electron density of the DRD2/haloperidol complex. a, b, 2Fo-Fc electron density map (blue mesh, contoured at 1.0 σ) of haloperidol (orange). **c, d,** Fo-Fc omit map (green mesh, contoured at 3.0 σ) of haloperidol. **e,** 2Fo-Fc electron density map of DRD2 binding pocket key residues (blue mesh) contoured at 1.0 σ. **f,** The salt bridge (dash line) between D114$^{3.32}$ and haloperidol. **g, h,** Top view of alignment between haloperidol/DRD2 (Orange stick and blue cartoon) and risperidone/DRD2 (yellow stick and green cartoon) complex. In all panels, receptors are shown as cartoon. Ligands and residues are shown as sticks. Ballesteros-Weinstein numbering is shown as superscript.

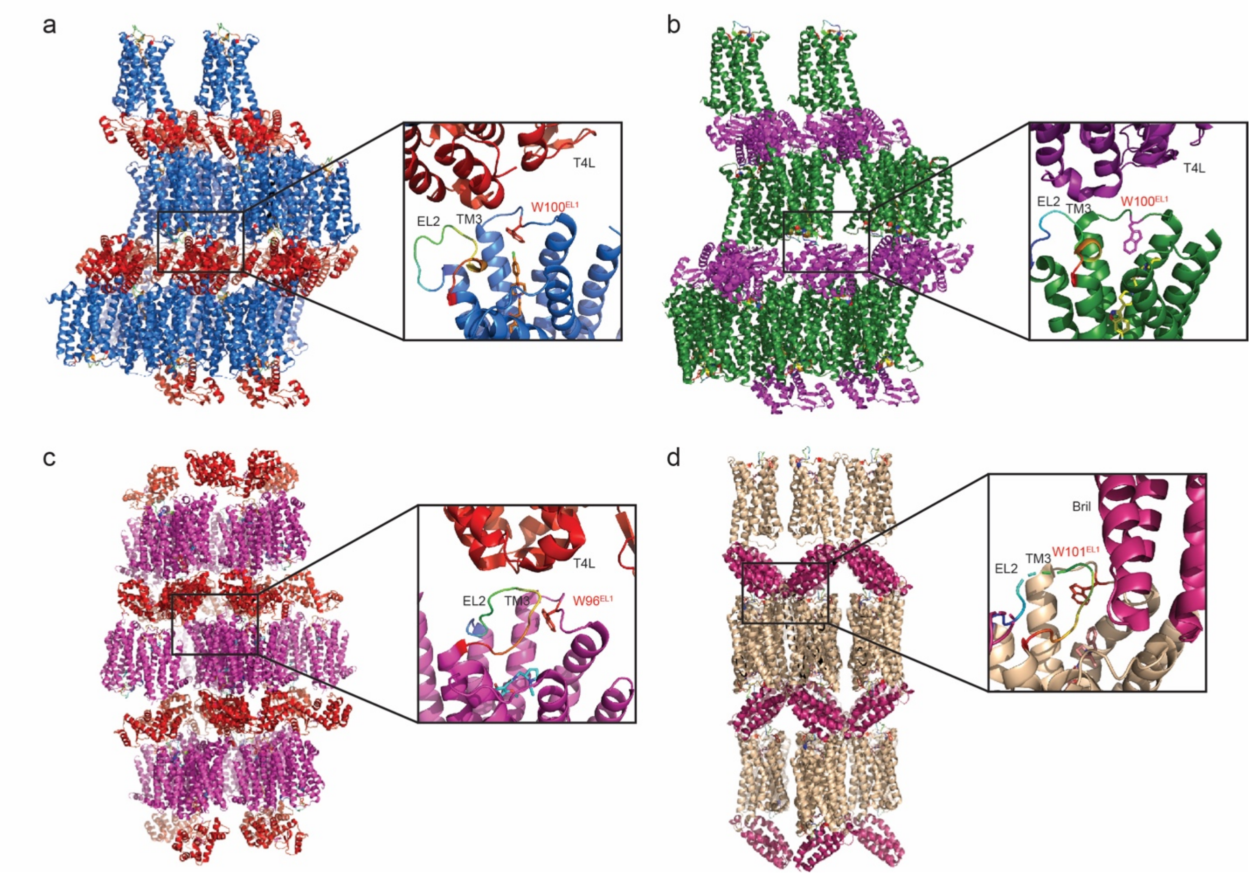

Supplementary Figure 2. Crystal packing of the D_2-like receptors. a, Packing of the DRD2/haloperidol complex. DRD2 is shown in blue and the T4L-fusion protein is shown in red. **b,** Packing of the DRD2/risperidone complex (PDB code 6CM4). DRD2 is shown in green and the T4L-fusion protein is shown in magenta. **c,** Packing of the DRD3/eticlopride complex (PDB code 3PBL). DRD3 is shown in pink and the T4L-fusion protein is shown in red. **d,** Packing of the DRD4/nemonapride complex (PDB code 5WIU). DRD4 is shown in wheat and the Bril-fusion protein is shown in magenta.

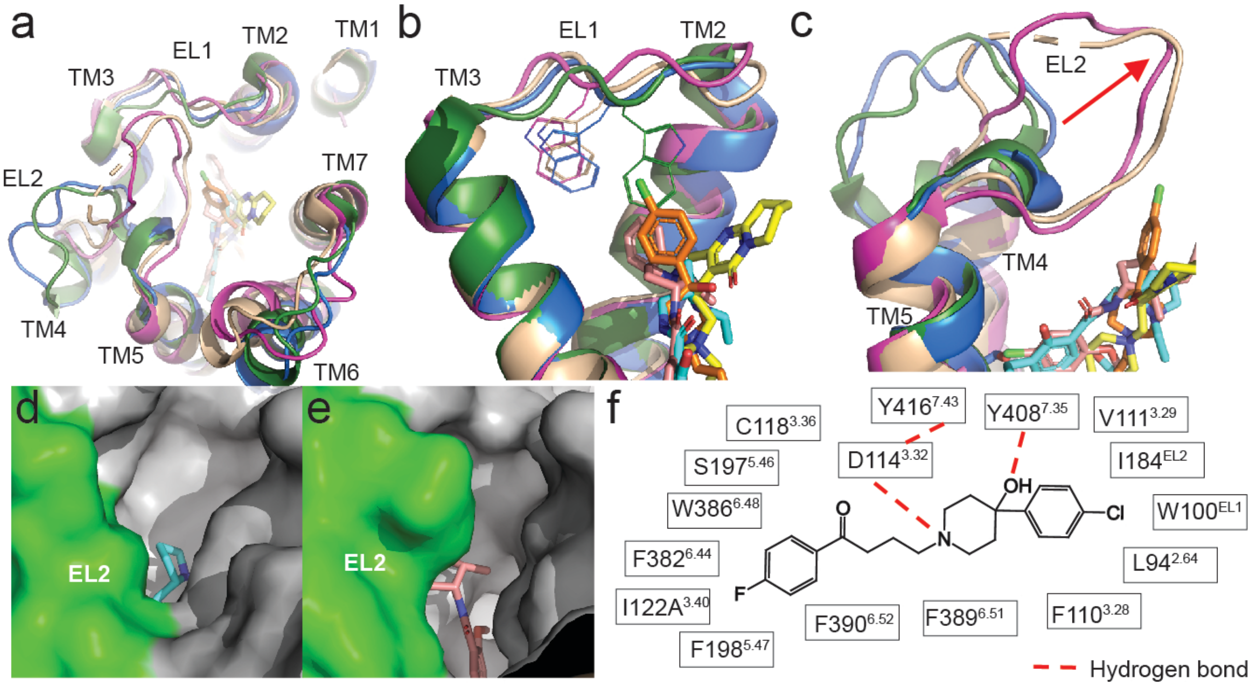

Supplementary Figure 3. Alignment of EL1 and EL2 of D$_2$-like receptors. In all panels, receptors are shown as cartoon or surface. Ligands and residues are shown as sticks. **a-c,** Superposition of conserved EL1 and EL2 at DRD2/haloperidol (blue cartoon/orange stick), DRD2/risperidone (green cartoon/yellow stick, PDB code 6CM4), DRD3/eticlopride (magenta cartoon/ cyan stick, PDB code 3PBL) and DRD4/nemonapride (tan cartoon/ pink stick, PDB code 5WIU). **d, e,** Top views of the ligand binding pocket in the DRD3/eticlopride (**d**) and DRD4/nemonapride (**e**) complexes. The pocket surface is colored gray except the EL2 in green. **f,** Diagram of ligand interactions in the binding pocket side chains at a 4.0-Å cutoff. Hydrogen bonds are shown with dashed lines.

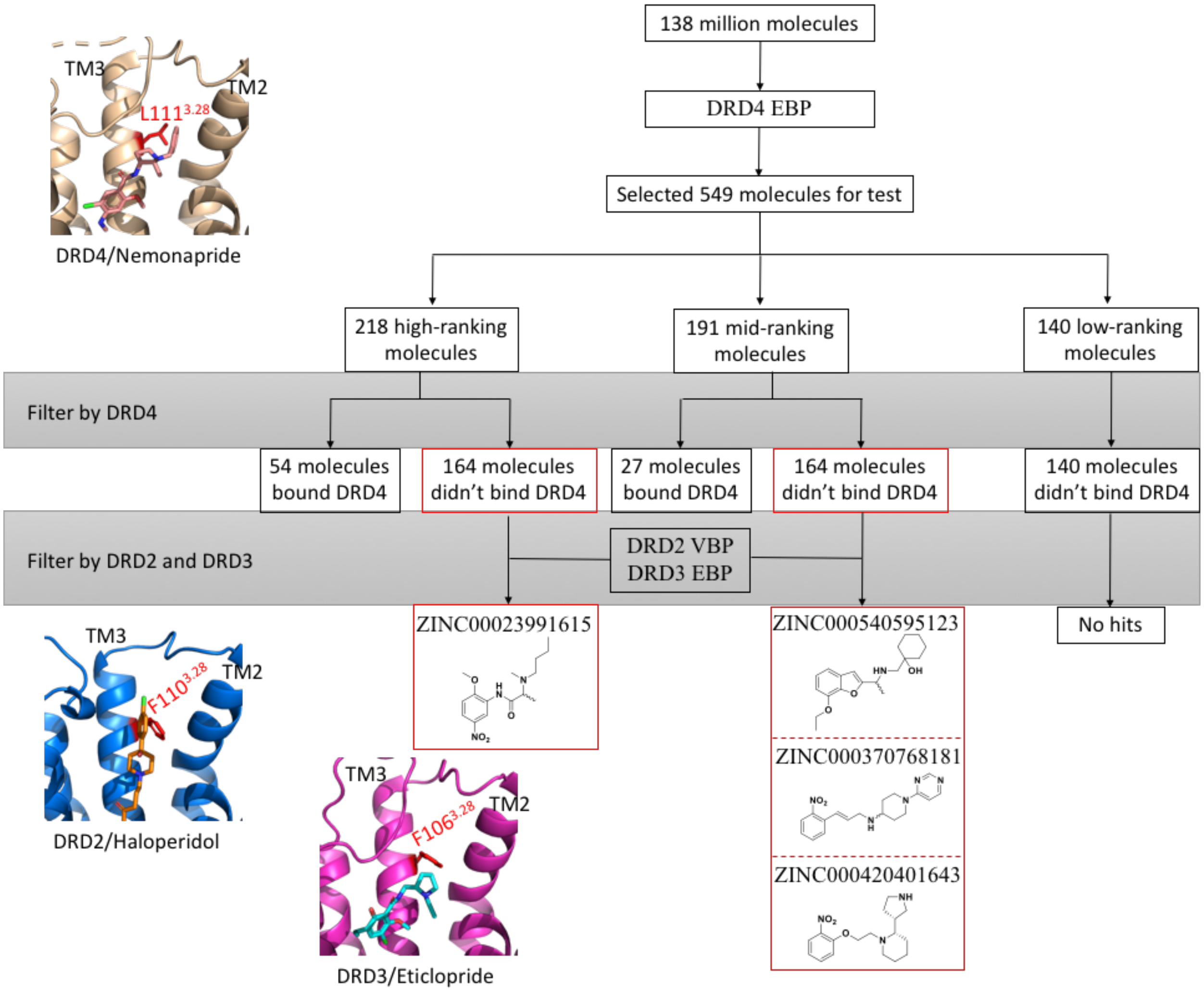

Supplementary Figure 4. Screening workflow. Receptors are shown as cartoon. Ligands and residues are shown as sticks. Ballesteros-Weinstein numbering is shown as superscript. The ranking reflects the docking scores over DRD4. The 549 molecules covered the highest-ranking (-75 to -63 kcal mol^{-1}), mid-ranking (-61 to -46 kcal mol^{-1}) and low-ranking scores (-43 to -35 kcal mol^{-1})[1]. See also Supplementary Table 3.

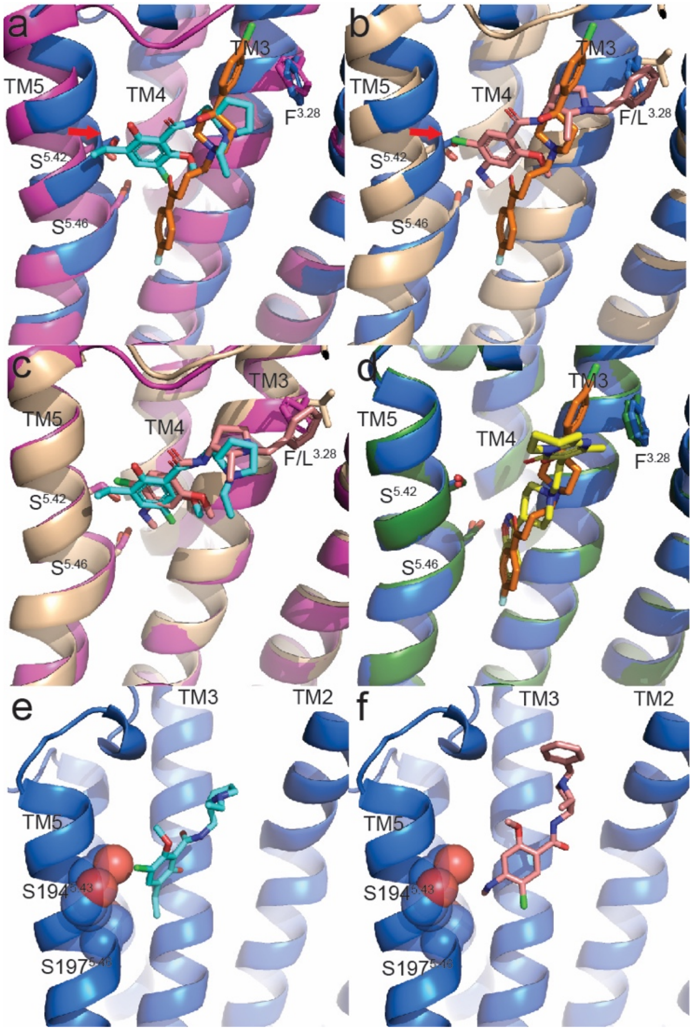

Supplementary Figure 5. Alignment of TM5 of D_2-like receptors and the docking pose of eticlopride/nemonapride at DRD2. In all panels, receptors are shown as cartoon. Ligands and residues are shown as sticks. **a, b, c, d,** Superposition of TM3, 4, 5 at DRD2/haloperidol ((blue cartoon/orange stick), DRD2/risperidone (green cartoon/yellow stick, PDB code: 6CM4), DRD3/eticlopride (magenta cartoon/cyan stick, PDB code: 3PBL) and DRD4/nemonapride (tan cartoon/pink stick, PDB code: 5WIU) aligned through TM1-4. **e, f,** Docking of eticlopride (cyan stick, **e**) and nemonapride (pink stick, **f**) in the DRD2 structure. Ballesteros-Weinstein numbering is shown as superscript. In all panels, receptors are shown as cartoon. Ligands and residues are shown as sticks.

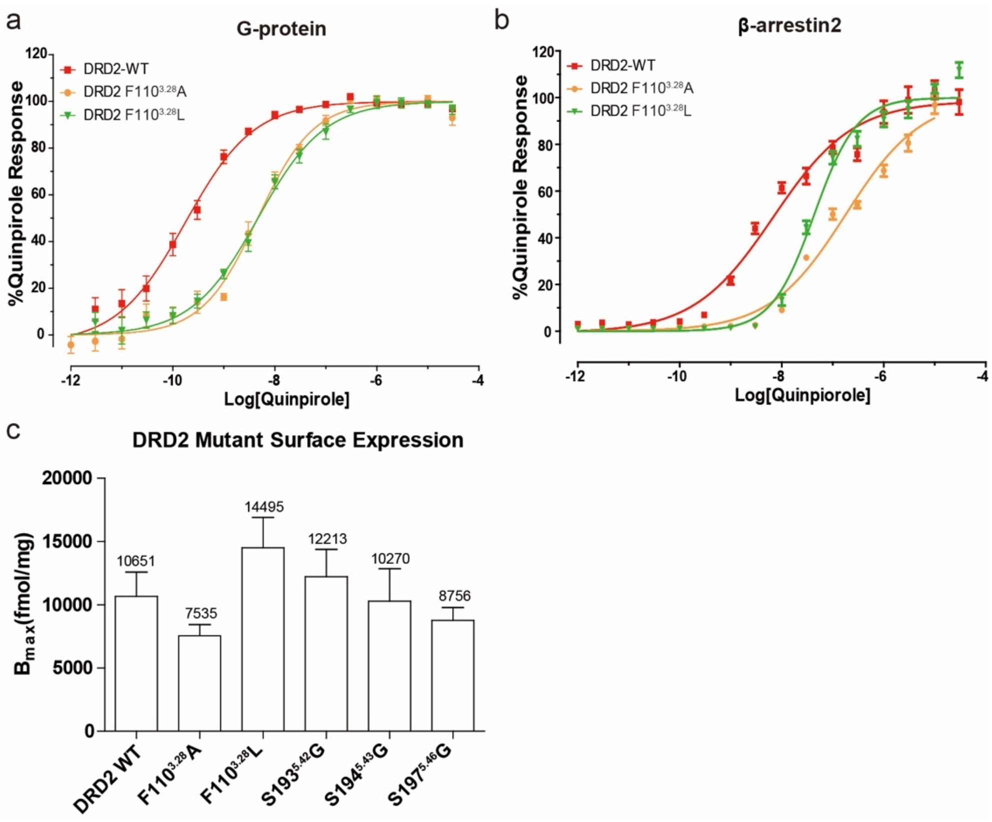

Supplementary Figure 6. Profiling of quinpirole at the mutations of Phe110$^{3.28}$ and the expression level of DRD2 mutants. a, b, Concentration-response studies for quinpirole in DRD2 or its mutants-mediated activation of G protein activity ($G_{\alpha i/o}$-mediated cAMP inhibition; **a**) and β-arrestin2 translocation (Tango; **b**). Data represent three independent experiments performed in triplicate technical replicates and in parallel using the same drug dilutions. Error bars, SEM (n = 3 independent experiments). **c**, The surface expression level of different DRD2 mutants. Measurement of saturation binding at different DRD2 mutants with ^{3}H-methylspiperone. Data represent means ± s.e.m. from three independent experiments (n = 3 independent experiments). Ballesteros-Weinstein numbering is shown as superscript. Source data are provided as a Source Data file.

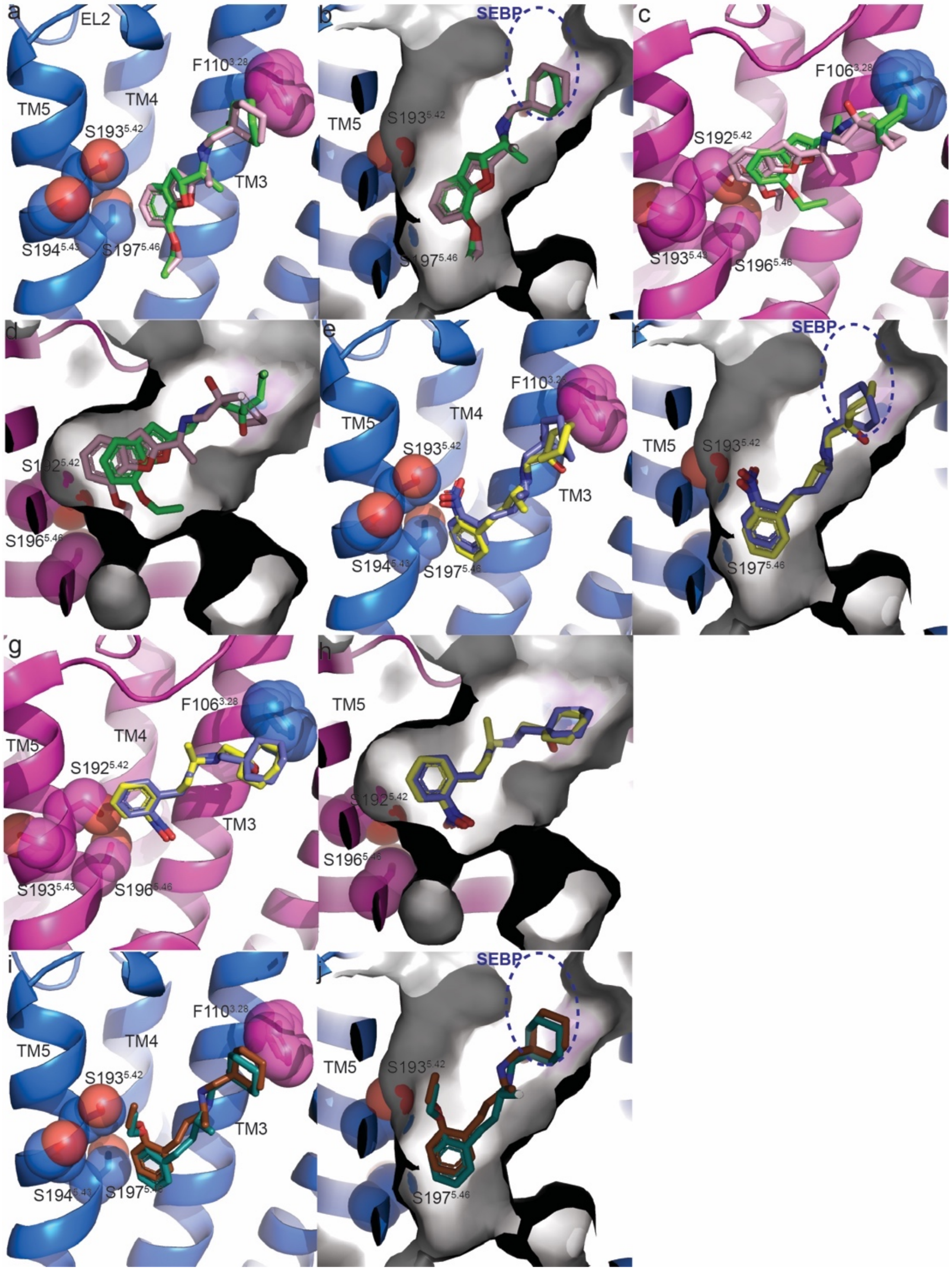

Supplementary Figure 7. Docking pose of O_4RE_6, O_4SE_6 , O_7RE_6, O_7SE_6, O_8RE_6 and O_8SE_6. In all panels, receptors are shown as cartoon or surface. Ligands are shown as sticks. Residues are shown as spheres. **a, b,** Docking pose of **O_4RE_6** (pink stick) and **O_4SE_6** (green stick) at the haloperidol-bound DRD2 crystal structure. **c, d,** Docking pose of **O_4RE_6** (pink stick) at eticlopride-bound DRD3 crystal structure. **e-h,** Docking pose of **O_7SE_6** (yellow stick) and **O_7RE_6** (slate stick) at haloperidol-bound DRD2 crystal structure (**e, f**) and eticlopride-bound DRD3 crystal structure (**g, h**). **i, j,** Docking pose of **O_8SE_6** (lime stick) and **O_8RE_6** (brown stick) at haloperidol-bound DRD2 crystal structure. Ballesteros-Weinstein numbering is shown as superscript. The position of SEBP is shown as an ellipse.

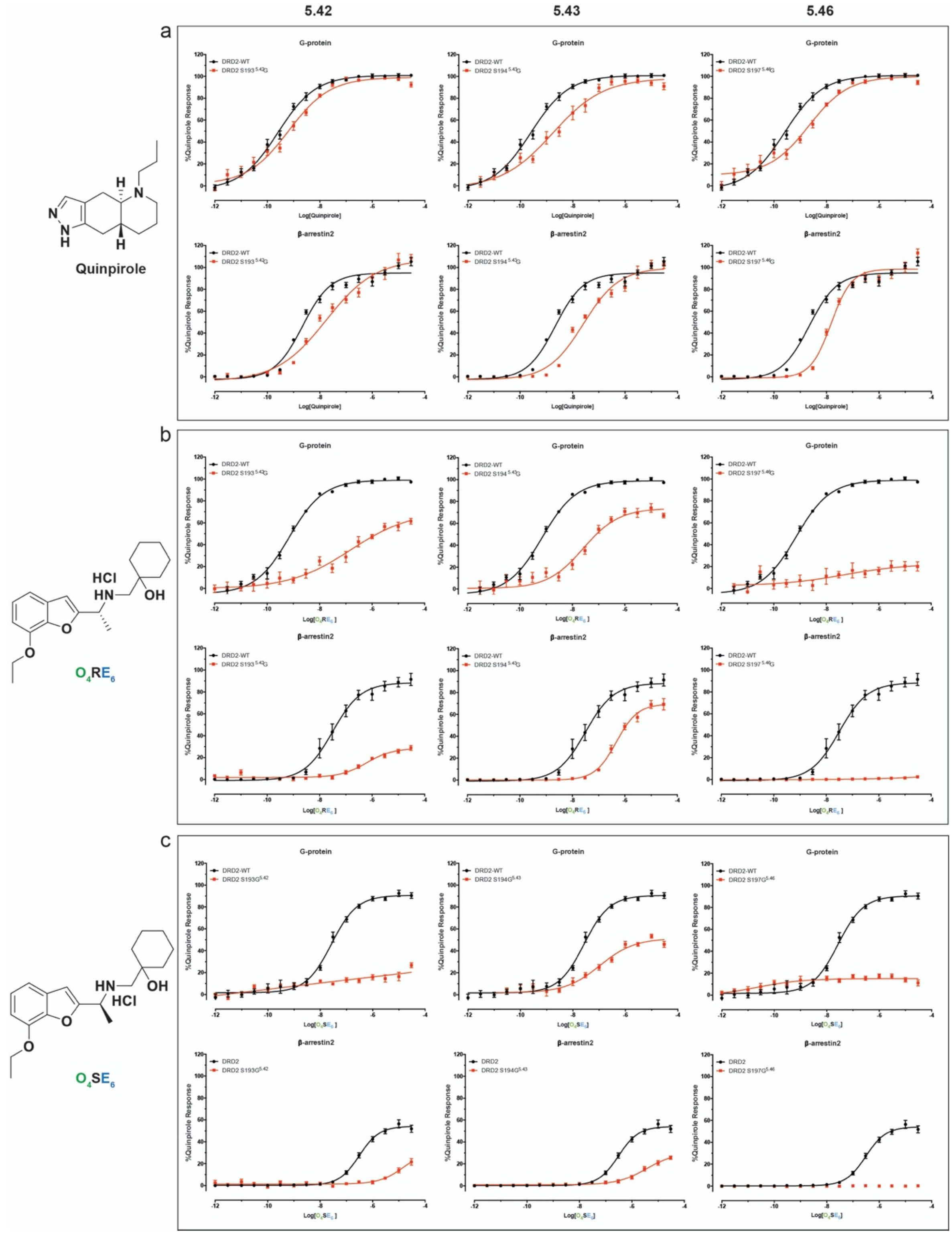

Supplementary Figure 8. Mutation of conserved TM5 serine decrease the O_4RE_6's and O_4SE_6's efficacy at DRD2. Profiling of quinpirole, **O_4RE_6** and **O_4SE_6** measuring DRD2 G protein activity ($G_{\alpha i/o}$-mediated cAMP inhibition) and β-arrestin2 recruitment (Tango), normalized to percent quinpirole activity. Data represent three independent experiments performed in triplicate technical replicates and in parallel using the same drug dilutions. Error bars, SEM (n = 3 independent experiments). Ballesteros-Weinstein numbering is shown as superscript. Source data are provided as a Source Data file.

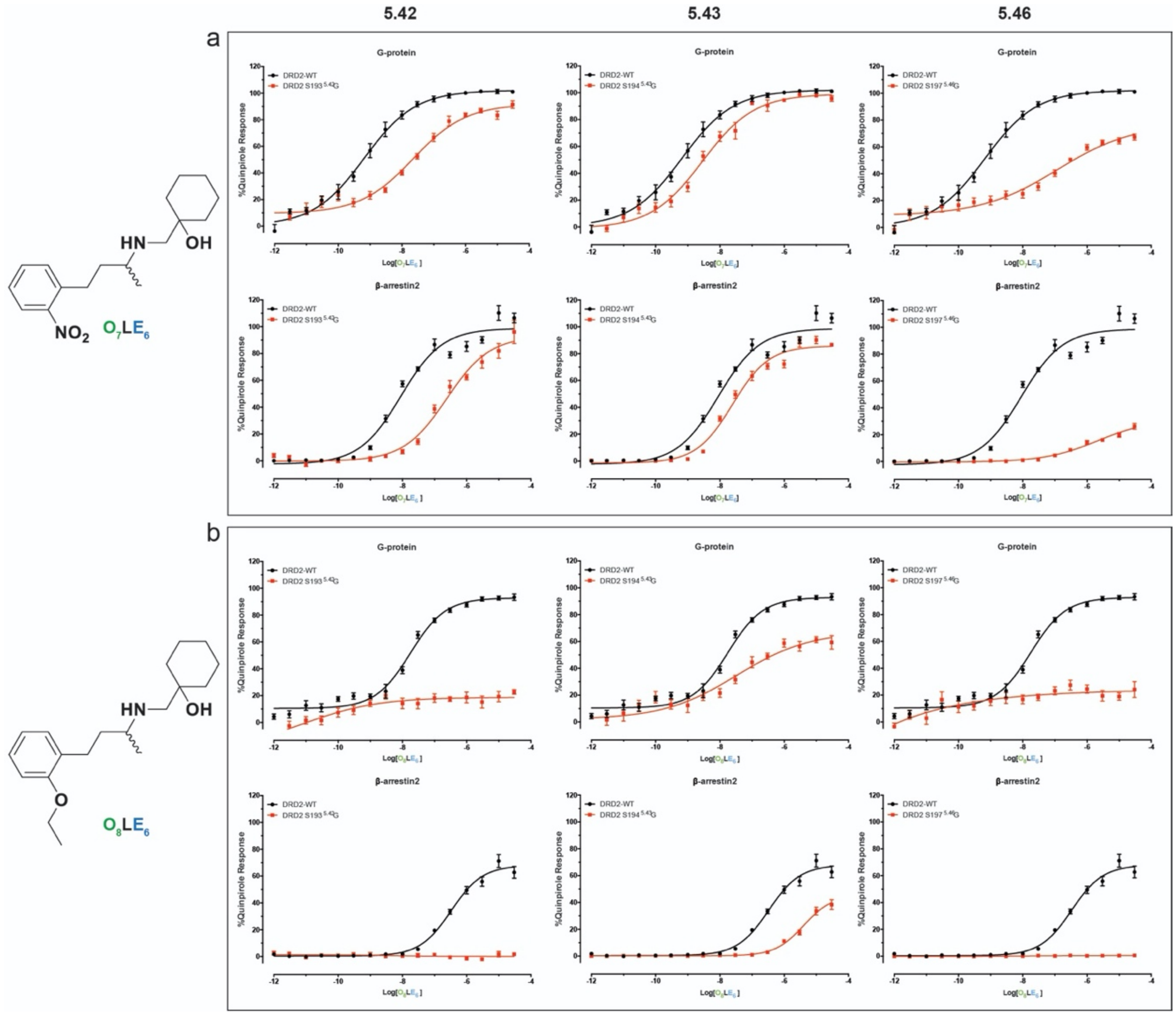

Supplementary Figure 9. Mutation of conserved TM5 serine decrease the O_7LE_6's and O_8LE_6's efficacy at DRD2. Profiling of quinpirole, **O_7LE_6** and **O_8LE_6** measuring DRD2 G protein activity ($G_{\alpha i/o}$-mediated cAMP inhibition) and β-arrestin2 recruitment (Tango), normalized to percent quinpirole activity. Data represent three independent experiments performed in triplicate technical replicates and in parallel using the same drug dilutions. Error bars, SEM (n = 3 independent experiments). Ballesteros-Weinstein numbering is shown as superscript. Source data are provided as a Source Data file.

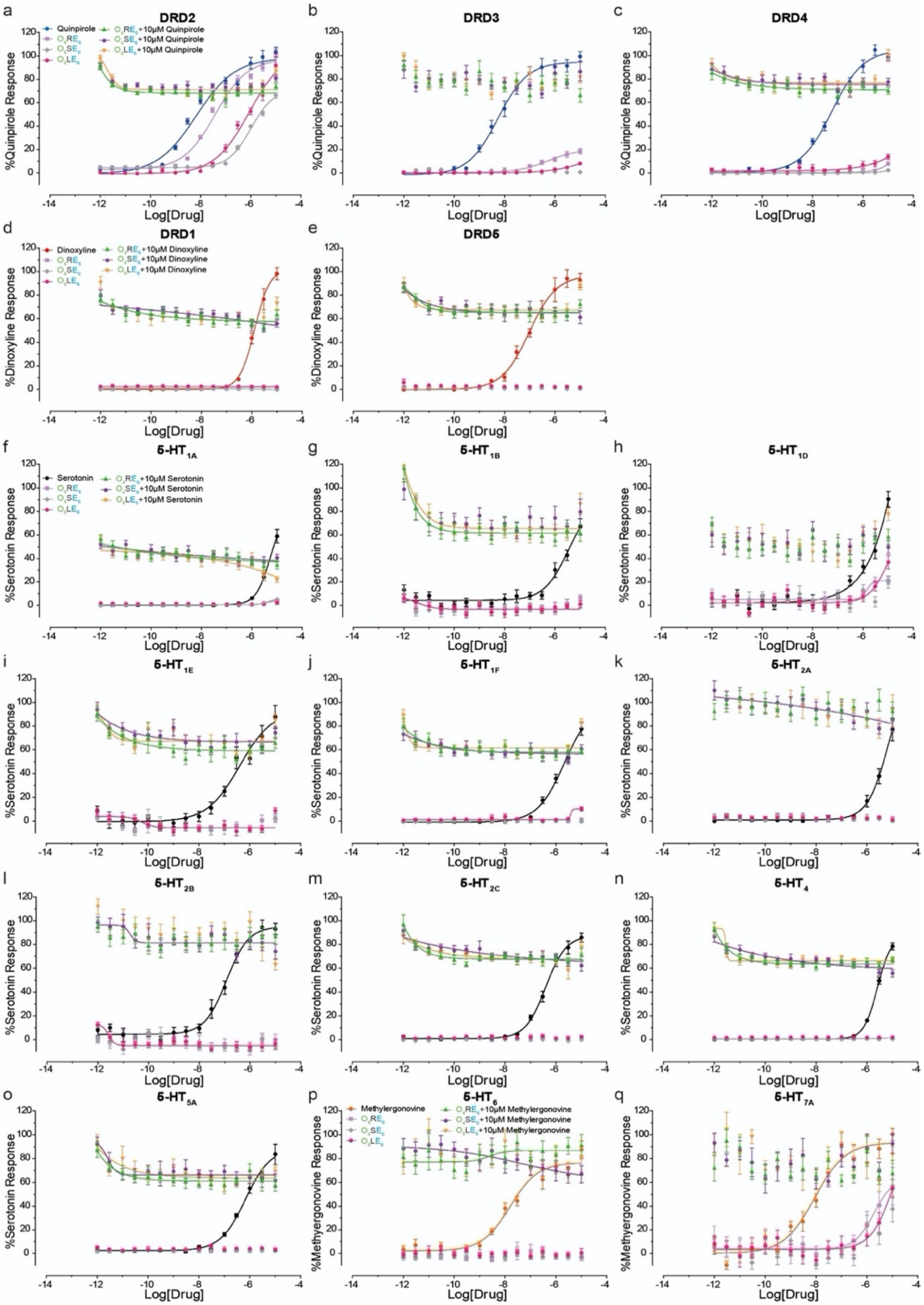

Supplementary Figure 10. Functional study of DRD2 selective ligands in dopamine and serotonin receptors. Profiling of **O_4SE_6**, **O_4RE_6** and **O_8LE_6** measuring β-arrestin2 recruitment (Tango) at dopamine and serotonin receptors, normalized to percent reference compounds activity. Data represent three independent experiments performed in triplicate technical replicates and in parallel using the same drug dilutions. Error bars, SEM (n = 3 independent experiments). Source data are provided as a Source Data file.

Supplementary Table 1. Data collection and refinement statistics (6LUQ)

	Human DRD2 (ΔN/ΔICL3$_{T4L}$/ΔC)-Haloperidol complex
**Data collection**	
Space group	$P2_12_12_1$
Cell dimensions	
a, *b*, *c* (Å)	50.43, 73.82, 150.27
α, β, γ (°)	90, 90, 90
Resolution (Å)	50.0 - 3.10 (3.20 - 3.10)*
R_{merge}	0.20(1.13)
$I / \sigma I$	7.3(1.2)
Completeness (%)	93.1(94.7)
Redundancy	4.1(4.0)
**Refinement**	
Resolution (Å)	50.0 - 3.10
No. reflections	9851
R_{work} / R_{free}	0.217 / 0.222
No. atoms	
Protein	3168
Ligand	26
Water	10
Lipid	80
B-factors (Å^2)	
Protein	80.9
Ligand	94.3
Water	59.7
Lipid	82.2
R.m.s. deviations	
Bond lengths (Å)	0.015
Bond angles (°)	1.75

*Values in parentheses are for highest-resolution shell.

Supplementary Table 2. Affinity of L-741626 and haloperidol at SEBP or EBP mutants of the D_2-like receptors.

Receptor	L-741626		Haloperidol	
	K_i, nM ($pK_i \pm$ SEM)	ΔpK_i (mutant-WT)	K_i, nM ($pK_i \pm$ SEM)	ΔpK_i (mutant-WT)
DRD2 wild-type	15.85 (7.80±0.15)	--	0.46 (9.34±0.09)	--
DRD2 L94$^{2.64}$A	44.67 (7.35±0.14)	-0.45	5.50 (8.26±0.07)	-1.08
DRD2 W100^{EL1}A	97.72 (7.01±0.26)	-0.79	1.51 (8.82±0.17)	-0.52
DRD2 W100^{ECL1}F	112.20 (6.95±0.33)	-0.85	13.18 (7.88±0.17)	-1.46
DRD2 V111$^{3.29}$A	28.84 (7.54±0.21)	-0.26	0.26 (9.59±0.02)	0.25
DRD2 Y408$^{7.35}$A	19.95 (7.70±0.13)	-0.10	0.60 (9.22±0.01)	-0.12
DRD2 F110$^{3.28}$A	0.11 (9.94±0.02)	2.14	0.03 (10.52±0.06)	1.18
DRD2 F110$^{3.28}$W	134.89 (6.87±0.39)	-0.93	12.88 (7.89±0.14)	-1.45
DRD2 F110$^{3.28}$Y	912.01 (6.04±0.44)	-1.76	19.05 (7.72±0.05)	-1.62
DRD2 F110$^{3.28}$L	0.85 (9.07±0.01)	1.27	0.26 (9.58±0.01)	0.24
DRD2 F110$^{3.28}$C	3.89 (8.41±0.47)	0.61	0.45 (9.35±0.21)	0.01
DRD2 F110$^{3.28}$E	14.79 (7.83±0.10)	0.03	0.79 (9.10±0.35)	-0.24
DRD3 wild-type	186.2 (6.73±0.07)	--	2.29 (8.64±0.08)	--
DRD3 F106$^{3.28}$A	5.37 (8.27±0.12)	1.54	1.15 (8.94±0.12)	0.30
DRD4 wild-type	169.8 (6.77±0.01)	--	1.82 (8.74±0.01)	--
DRD4 L111$^{3.28}$F	346.7 (6.46±0.01)	-0.31	19.49 (7.71±0.02)	-1.03

Data represent mean K_i ($pK_i \pm$ SEM) for competition binding experiments using [^{3}H]-methylspiperone (0.8-1.0 nM) as radioligand. All data are the mean $\pm$ SEM of three independent assays (n = 3 independent experiments).

Supplementary Table 3. Docking hits tested at DRD2/DRD3/DRD4.

Cmpd	ZINC ID	Structure	DRD2 EC_{50}± SEM, nM	K_i, µM (pK_i ± SEM)		
				DRD2	DRD3	DRD4
O_9LE_9	ZINC00023991615	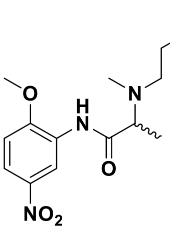	3555±295 (antagonist, G$\alpha_{i/o}$-mediated cAMP inhibition)	3.80 (5.42±0.05)	0.21 (6.67±0.02)	>10
O_4LE_6	ZINC000540595123	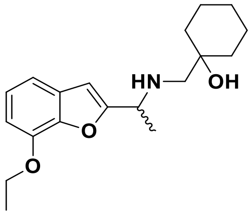	0.57±0.07 (agonist, G$\alpha_{i/o}$-mediated cAMP inhibition)<br><br>24.14±7.57 (agonist, Gαi1-γ2 dissociation, BRET)	1.91 (5.72±0.12)	0.22 (6.65±0.14)	>10
O_7LE_{10}	ZINC000370768181	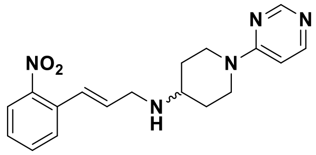	NT*	>10	2.75 (5.56±0.01)	>10
O_7LE_{11}	ZINC000420401643	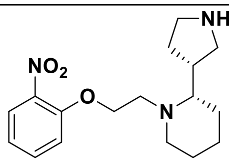	NT	>10	4.27 (5.37±0.10)	>10
Dopamine		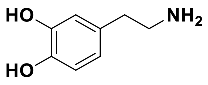	0.20±0.06 (agonist, G$\alpha_{i/o}$-mediated cAMP inhibition)<br><br>50.37±6.97 (agonist, Gαi1-γ2 dissociation, BRET)	1.58 (5.80±0.08)	0.34 (6.47±0.01)	0.08 (7.11±0.06)

Data represent mean EC_{50}± SEM or K_i (pK_i ± SEM) for competition binding experiments using [^{3}H]-methylspiperone (0.8-1.0 nM) as radioligand. All data are the mean ± SEM of three independent assays (n = 3 independent experiments). *NT: Not tested.

Supplementary Table 4. Affinity of OLE analogs at D_2-like dopamine receptors and its mutations.

Cmpd	Structure	K_i, µM (pK_i ± SEM)				
		DRD2	DRD3	DRD4	DRD2 F110$^{3.28}$A	DRD3 F106$^{3.28}$A
O$_4$LE$_5$	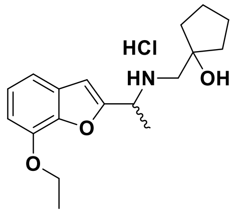	9.55 (5.02±0.07)	1.02 (5.99±0.02)	>10	0.93 (6.03±0.14)	4.27 (5.37±0.13)
O$_4$LE$_6$	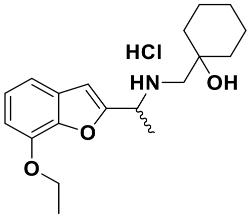	1.90 (5.72±0.12)	0.22 (6.65±0.14)	>10	0.72 (6.14±0.11)	0.46 (6.34±0.10)
O$_4$LE$_7$	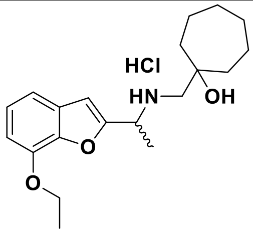	1.58 (5.80±0.04)	0.24 (7.62±0.22)	4.07 (5.39±0.04)	0.21 (6.67±0.16)	0.42 (6.38±0.03)
O$_4$LE$_8$	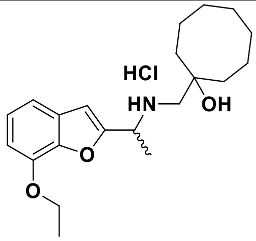	1.44 (5.84±0.10)	0.13 (6.90±0.20)	3.47 （5.46±0.10）	0.17 (6.76±0.03)	0.21 (6.68±0.02)
O$_4$RE$_6$	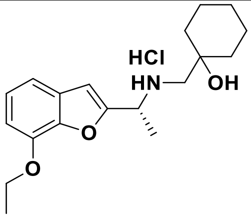	0.69 (6.16±0.18)	0.11 (6.94±0.12)	>10	0.49 (6.31±0.17)	1.05 (5.98±0.09)
O$_4$SE$_6$	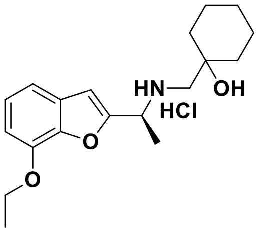	4.57 (5.34±0.13)	0.81 (6.09±0.20)	>10	0.52 (6.28±0.12)	1.91 (5.72±0.22)

O_7LE_6	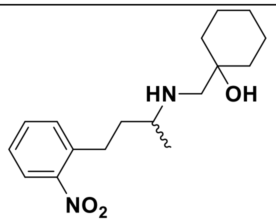	1.41 (5.85±0.25)	0.17 (6.76±0.21)	>10	NT*	NT
O_8LE_6	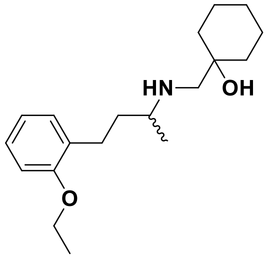	1.86 (5.73±0.34)	0.43 (6.37±0.16)	>10	NT	NT

Data represent mean K_i (pK_i ± SEM) for competition binding experiments using $[^3H]$-methylspiperone (0.8-1.0 nM) as radioligand. All data are the mean ± SEM of three independent assays (n = 3 independent experiments). *NT: Not tested.

Supplementary Table 5. G$\alpha_{i/o}$-mediated cAMP inhibition signaling of OLE analogs at D_2-like dopamine receptors and its mutations.

Cmpd	Structure	EC_{50}± SEM, nM				
		DRD2	DRD3	DRD4	DRD2 F110$^{3.28}$A	DRD2 F106$^{3.28}$L
Quinpirole	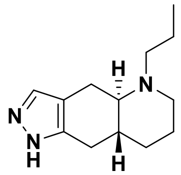	0.20±0.11<br>(100.0%)	0.23±0.08<br>(100.0%)	0.17±0.04<br>(100.0%)	3.39±0.02<br>(100.0%)	4.04±0.04<br>(100.0%)
O_4LE_5	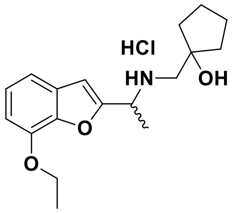	20.33±9.06<br>（90.02%）	NT	>10000	0.05±0.04<br>（13.66%）	>10000
O_4LE_6	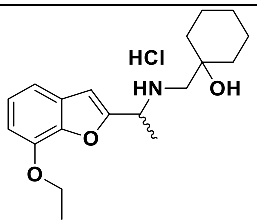	0.57±0.07<br>（94.81%）	5.09±0.16<br>（62.44%）	>10000	>10000	11.27±2.27<br>（61.00%）
O_4LE_7	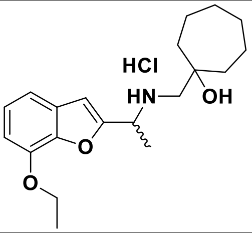	1.65±1.05<br>（95.25%）	NT	>10000	>10000	3.61±0.07<br>（72.59%）
O_4LE_8	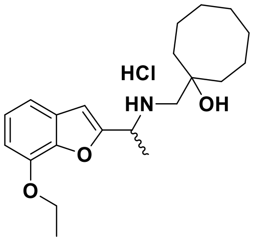	25.13±4.15<br>（93.50%）	NT	>10000	>10000	5.14±1.34<br>（79.40%）
O_4RE_6	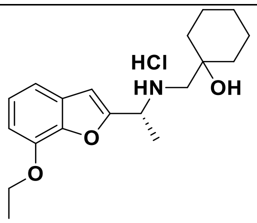	0.45±0.05<br>(94.46%)	7.12±0.08<br>(52.44%)	>10000	>10000	1.24±0.75<br>(56.42%)

O_4SE_6	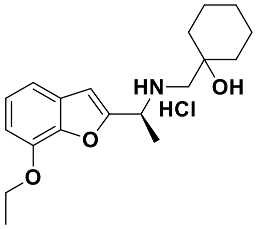	18.45±0.37 (91.92%)	>10000	>10000	>10000	>10000
O_7LE_6	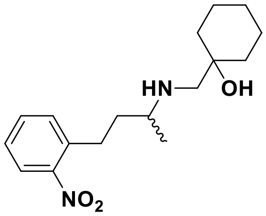	1.13±0.49 (99.98%)	1.96±0.54 (82.68%)	>10000	NT**	NT
O_8LE_6	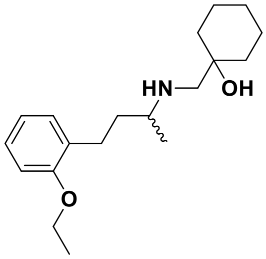	30.42±10.89 (89.76%)	>10000	>10000	NT	NT

All data are the mean ± SEM of three independent assays (n = 3 independent experiments). *The percentage of E_{max} relative to quinpirole response. **NT: Not tested.

Supplementary Table 6. β-arrestin signaling of OLE analogs at D_2-like dopamine receptors and its mutations.

Cmpd	Structure	EC$_{50}$± SEM, nM					
		DRD2	DRD3	DRD4	DRD2 F110$^{3.28}$A	DRD2 F110$^{3.28}$L	DRD3 F106$^{3.28}$A
Quinpirole	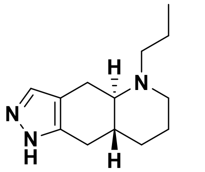	7.34±0.18 (100%)	7.54±0.11 (100%)	54.39±0.45 (100%)	210.20±66.4 (100%)	44.17±6.67 (100%)	NT
O_4LE_5	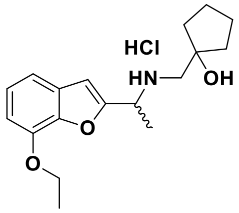	591.1±160.3（69.77%）	>10000	>10000	>10000	>10000	>10000
O_4LE_6	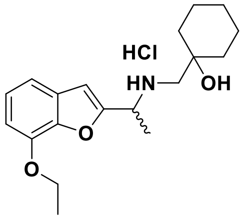	43.21±12.35（104.90%）	708.5±6.3（15.78%）	>10000	>10000	203.9±22.6 (4.32%)	>10000
O_4LE_7	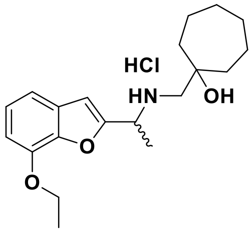	44.64±4.71（71.79%）	803.1±264.6（32.28%）	>10000	>10000	504.3±346.3 (25.78%)	>10000
O_4LE_8	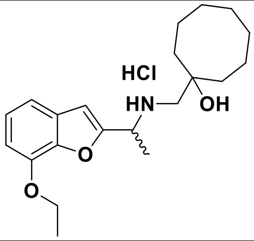	958.1±554.1（53.97%）	2569±331（12.56%）	7725±3424（22.28%）	>10000	83.24±18.43（12.90%）	>10000
O_4RE_6	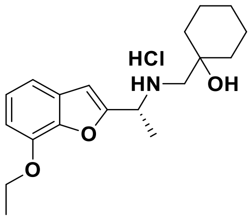	48.80±9.47 (99.61%)	808.4±22.0 (21.41%)	>10000	>10000	215.1±62.6 (7.76%)	NT

**O$_4$SE$_6$**	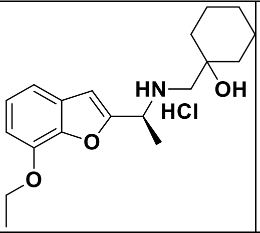	1055±178.9 (71.33%)	>10000	>10000	>10000	>10000	NT
**O$_7$LE$_6$**	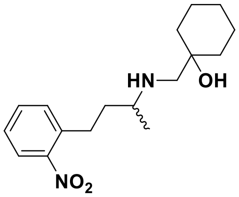	6.95±2.53 （85.33%）	117.1±21.6 （88.46%）	>10000	NT**	NT	NT
**O$_8$LE$_6$**	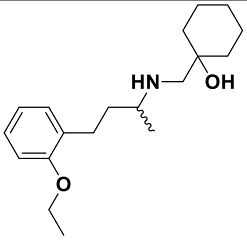	311.0±146.6 （63.81%）	>10000	>10000	NT	NT	NT

All data are the mean ± SEM of three independent assays (n = 3 independent experiments). *The percentage of E_{max} relative to quinpirole response. **NT: Not tested.

Supplementary Note 1: Structure-activity relationship of OLE compounds

To study the structure-activity relationships (SARs) of compound **O_4LE_6**, we designed and synthesized 14 analogs of this lead compound, with particular focus on the ethoxy substituent on the left-hand side (LHS) benzofuran part which supposedly occupies the DRD2 OBP, and the right-hand side (RHS) cyclohexane moiety which likely binds to the SEBP and contacts with the residue Phe110$^{3.28}$, respectively (Supplementary Fig. 11). For the 6 analogs (**$O_{1\text{-}6}LE_6$**) with various substitutions on the LHS benzofuran, the most potent analogs are **O_4** and **O_5** compounds, which have ethoxy and propoxy substituents respectively (based on the EC_{50}s of $G\alpha_{i/o}$ agonism and β-arrestin2 recruitment assays) (Supplementary Fig. 11b, c, Supplementary Table 4-9). For the RHS cyclohexane part (**$O_4LE_{1\text{-}9}$**), we found that the best substituents are cyclohexane (**E_6**) and cycloheptane (**E_7**) (based on the EC_{50}s of $G\alpha_{i/o}$ agonism and β-arrestin2 recruitment assays) (Supplementary Fig. 11b, c, Supplementary Table 4-9).

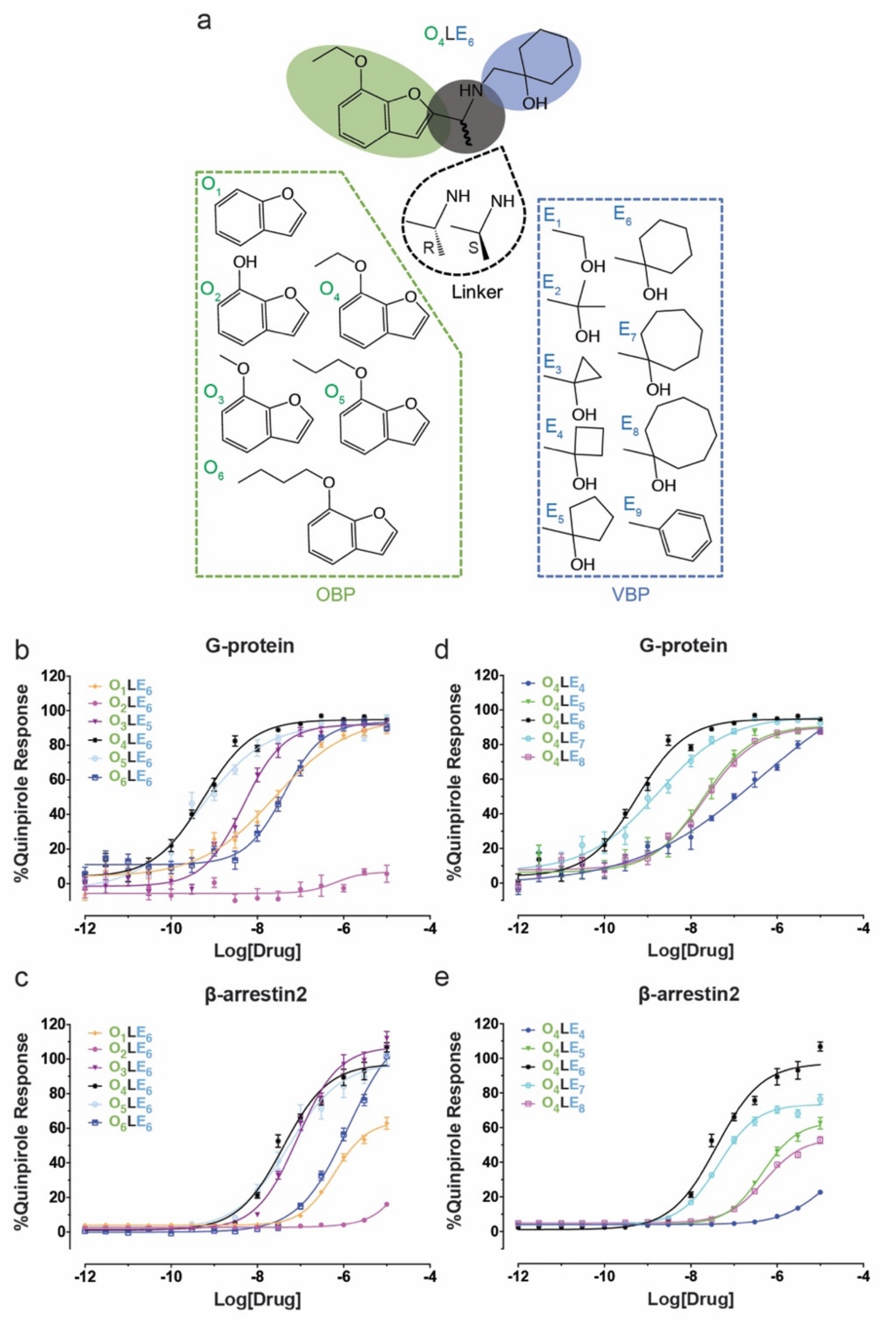

Supplementary Figure 11. Structure-activity relationship of OLE compounds. a, The analogs of **OLE** compounds. **b-e**, Concentration-response studies for analogs of **OLE** in DRD2-mediated activation of G protein activity ($G_{\alpha i/o}$-mediated cAMP inhibition; **b**, **d**) and β-arrestin2 translocation (Tango; **c**, **e**), normalized to percent quinpirole activity. Data represent three independent experiments performed in triplicate technical replicates and in parallel using the same drug dilutions. Error bars, SEM (n = 3 independent experiments). See also Supplementary Table 5 and 6. Source data are provided as a Source Data file.

Supplementary Table 7. Affinity of OLE analogs at D_2-like dopamine receptors and its mutations.

Cmpd	Structure	K_i, μM (pK_i ± SEM)				
		DRD2	DRD3	DRD4	DRD2 F110$^{3.28}$A	DRD3 F106$^{3.28}$A
O_1LE_6	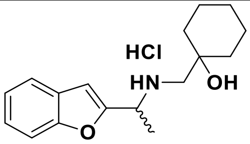	>10	0.76 (6.12±0.17)	5.89 (5.23±0.05)	>10	NT*
O_2LE_6	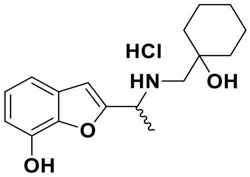	>10	3.47 (5.46±0.10)	>10	5.75 (5.24±0.17)	NT
O_3LE_6	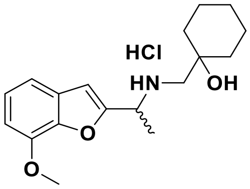	4.78 (5.32±0.22)	1.07 (5.97±0.13)	>10	3.24 (5.49±0.16)	NT
O_5LE_6	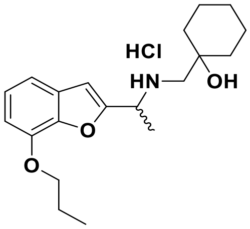	2.51 (5.60±0.27)	0.39 (6.41±0.20)	6.60 (5.18±0.16)	0.68 (6.17±0.24)	NT
O_6LE_6	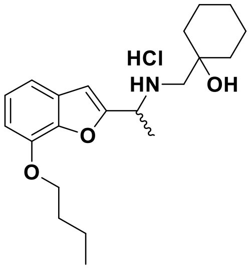	2.04 (5.69±0.06)	0.85 (6.07±0.01)	>10	0.30 (6.53±0.31)	NT
O_4LE_9	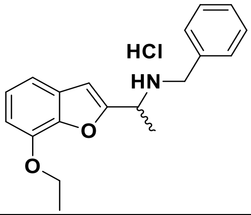	>10	5.88 (5.23±0.25)	>10	5.25 (5.28±0.02)	NT
O_4LE_1	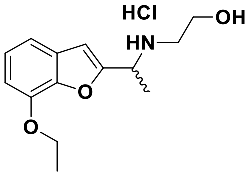	>10	>10	>10	>10	>10

O_4LE_2	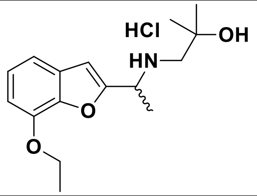	>10	>10	>10	>10	>10
O_4LE_3	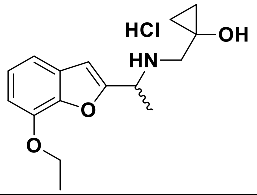	>10	4.37 (5.36±0.31)	>10	>10	>10
O_4LE_4	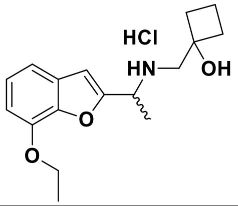	>10	4.07 (5.39±0.09)	>10	>10	>10

Data represent mean K_i (pK_i ± SEM) for competition binding experiments using [^{3}H]-methylspiperone (0.8-1.0 nM) as radioligand. All data are the mean ± SEM of three independent assays (n = 3 independent experiments). *NT: Not tested.

Supplementary Table 8. G$\alpha_{i/o}$-mediated cAMP inhibition signaling of OLE analogs at D_2-like dopamine receptors and its mutations.

Cmpd	Structure	EC_{50}± SEM, nM				
		DRD2	DRD3	DRD4	DRD2 F110$^{3.28}$A	DRD2 F110$^{3.28}$L
O_1LE_6	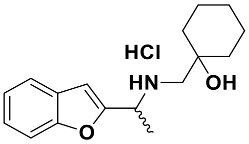	33.15±18.77 (91.90%)*	NT**	>10000	NT	NT
O_2LE_6	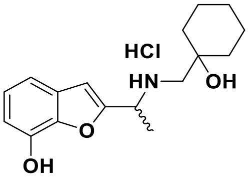	>10000	NT	>10000	NT	NT
O_3LE_6	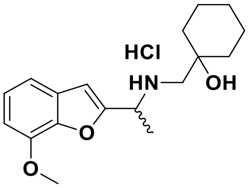	5.38±1.15 （91.62%）	NT	>10000	NT	NT
O_5LE_6	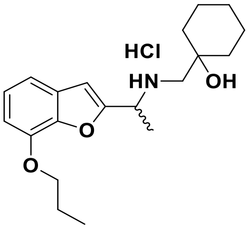	0.51±0.22 （92.80%）	NT	>10000	NT	NT
O_6LE_6	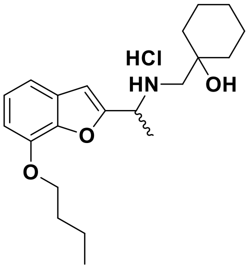	46.42±15.45 (96.65%)	NT	>10000	NT	NT
O_4LE_9	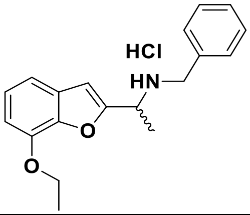	>10000	NT	>10000	NT	NT

O_4LE_1	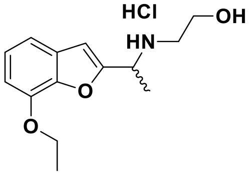	>10000	NT	>10000	NT	NT
O_4LE_2	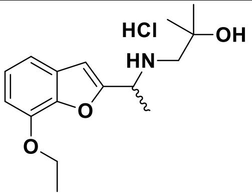	>10000	NT	>10000	NT	NT
O_4LE_3	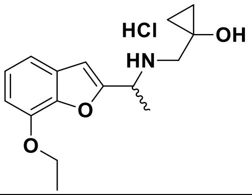	>10000	NT	>10000	>10000	>10000
O_4LE_4	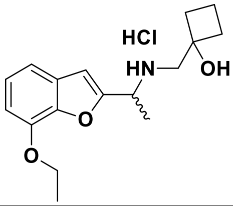	>10000	NT	>10000	>10000	>10000

All data are the mean ± SEM of three independent assays (n = 3 independent experiments). *The percentage of E_{max} relative to quinpirole response. **NT: Not tested.

Supplementary Table 9. β-arrestin signaling of OLE analogs at D_2-like dopamine receptors and its mutations.

Cmpd	Structure	EC_{50}± SEM, nM					
		DRD2	DRD3	DRD4	DRD2 F110$^{3.28}$A	DRD2 F110$^{3.28}$L	DRD3 F106$^{3.28}$A
O_1LE_6	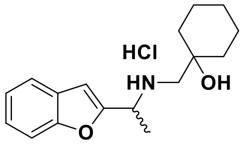	797.4±57.68 (70.08%)*	2530±290 （32.40%）	>10000	NT**	NT	NT
O_2LE_6	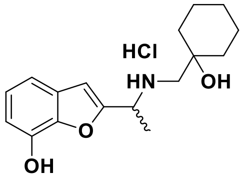	>10000	>10000	>10000	NT	NT	NT
O_3LE_6	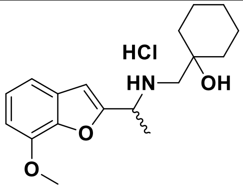	166.3±76.4 （109.80%）	5476±3943 （80.01%）	>10000	NT	NT	NT
O_5LE_6	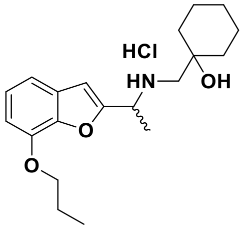	47.96±17.38 （96.83%）	2074±524 (53.92%)	>10000	NT	NT	NT
O_6LE_6	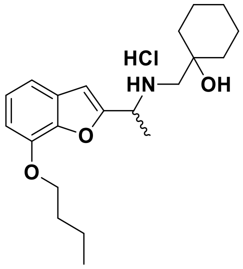	557.3±128.6 (83.11%)	>10000	>10000	NT	NT	NT
O_4LE_9	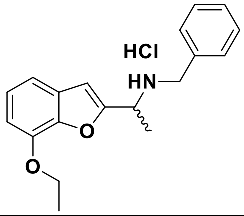	5368±964 （19.73%）	>10000	>10000	NT	NT	NT
O_4LE_1	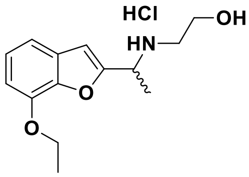	>10000	>10000	>10000	NT	NT	NT

O_4LE_2	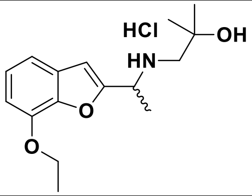	>10000	>10000	>10000	NT	NT	NT
O_4LE_3	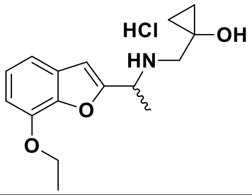	8850 ±2621 （9.29%）	>10000	>10000	>10000	>10000	>10000
O_4LE_4	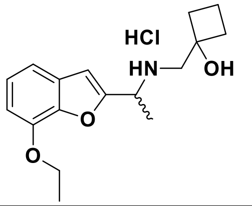	4889±671 （31.72%）	>10000	>10000	>10000	>10000	>10000

All data are the mean ± SEM of three independent assays (n = 3 independent experiments). *The percentage of E_{max} relative to quinpirole response. **NT: Not tested.

Supplementary Note 2: Synthetic Chemistry Procedures

Synthesis of compound O_1LE_6:

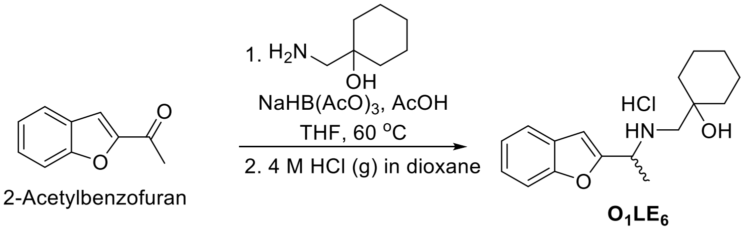

1-(((1-(Benzofuran-2-yl)ethyl)amino)methyl)cyclohexan-1-ol Hydrochloride (O_1LE_6). A mixture of 2-acetylbenzofuran (120 mg, 0.75 mmol), 1-(aminomethyl)cyclohexanol (116 mg, 0.90 mmol) and AcOH (90 mg, 1.5 mmol) in anhydrous THF (15 mL) was stirred at 60 °C for 24 h. $NaHB(AcO)_3$ (317 mg, 1.5 mmol) was added and the resulting mixture was stirred at 60 °C for 2 h. After being cooled to room temperature, methanol (5 mL) was added and the reaction mixture was stirred for 10 min. The reaction mixture was then concentrated and the residue was purified by flash chromatography (0 – 5% methanol in dichloromethane) to give a colorless oil (52 mg, 25% yield). The oil was dissolved in a mixture of methanol and dichloromethane (1:10, 5 mL) and then treated with 4 *M* HCl (g) in dioxane (1 mL). Removal of volatiles gave the title compound **O_1LE_6** as a white solid (58 mg, 98% yield). HPLC: 98.6%, t_R = 12.7 min; ^{1}H NMR (800 MHz, CD_3OD) δ 7.66 (d, J = 7.8 Hz, 1H), 7.55 (d, J = 8.3 Hz, 1H), 7.39 – 7.36 (m, 1H), 7.29 (t, J = 7.5 Hz, 1H), 7.08 (s, 1H), 4.78 (q, J = 6.9 Hz, 1H), 2.92 and 2.88 (ABq, J = 12.8 Hz, 2H), 1.82 (d, J = 7.0 Hz, 3H), 1.67 – 1.57 (m, 4H), 1.55 – 1.39 (m, 5H), 1.32 – 1.26 (m, 1H). ^{13}C NMR (201 MHz, CD_3OD) δ 156.7, 152.3, 128.8, 126.7, 124.6, 122.8, 112.4, 109.2, 69.4, 55.2, 53.4, 36.2 (2C), 26.4, 22.6 (2C), 16.1. HRMS (*m/z*): $[M + H]^+$ calculated for $C_{17}H_{24}NO_2^+$, 274.1802; found, 274.1806.

Synthesis of compound O_2LE_6:

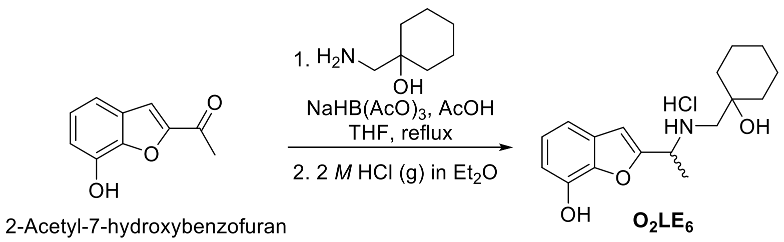

2-(1-(((1-Hydroxycyclohexyl)methyl)amino)ethyl)benzofuran-7-ol Hydrochloride (O_2LE_6). A mixture of 2-acetyl-7-hydrobenzofuran (80 mg, 0.454 mmol), 1-(aminomethyl)cyclohexanol (59 mg, 0.454 mmol) and AcOH (27 mg, 0.454 mmol) in anhydrous THF (10 mL) was stirred at 60 °C for 1 h. $NaHB(AcO)_3$ (193 mg, 0.908 mmol) was added and the resulting mixture was stirred at room temperature overnight. The reaction mixture was diluted with water and extracted with ethyl acetate twice. The combined extracts were washed with brine and concentrated in vacuum to give a residue. The residue was purified by flash chromatography (0 – 7% methanol in dichloromethane) to give a white solid (26 mg, 20% yield). The solid was dissolved in a mixture of methanol and dichloromethane (1:10, 5 mL) and then treated with 2 *M* HCl (g) in diethyl ether (2 mL). Removal of volatiles gave the title compound **O_2LE_6** as a white solid (24 mg, 82% yield). HPLC: 98.3%, t_R = 11.2 min; ^1H NMR (600 MHz, CD_3OD) δ 7.02 – 6.96 (m, 2H), 6.91 (s, 1H), 6.70 (dd, J = 7.3, 1.5 Hz, 1H), 4.65 (q, J = 7.0 Hz, 1H), 2.84 – 2.78 (m, 2H), 1.71 (d, J = 7.0 Hz, 3H), 1.57 – 1.47 (m, 4H), 1.46 – 1.29 (m, 5H), 1.24 – 1.19 (m, 1H). ^{13}C NMR (201 MHz, CD_3OD) δ 152.1, 145.6, 144.0, 130.7, 125.4, 113.6, 112.5, 109.6, 69.5, 55.3, 53.4, 36.2 (2C), 26.4, 22.6 (2C), 16.1. HRMS (*m/z*): $[M + H]^+$ calculated for $C_{17}H_{24}NO_3^+$, 290.1751; found, 290.1749.

Syntheses of compounds O_3LE_6 ~ O_6LE_6:

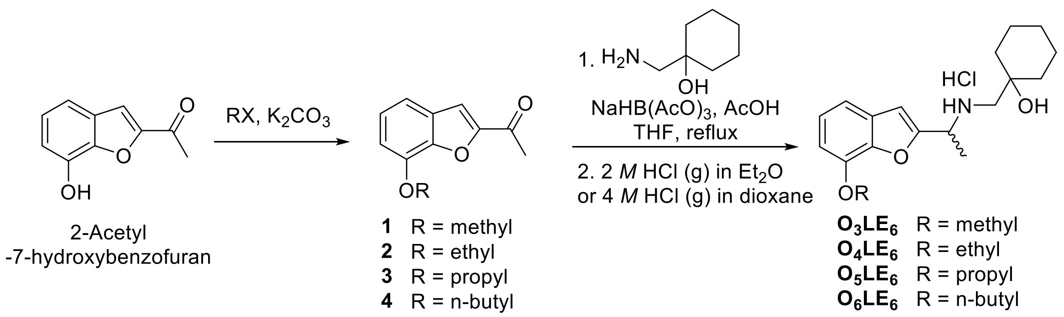

1-(7-Methoxybenzofuran-2-yl)ethan-1-one (1). A mixture of 2-acetyl-7-hydrobenzofuran (200 mg, 1.1 mmol), iodomethane (242 mg, 1.7 mmol) and K_2CO_3 (314 mg, 2.3 mmol) in DMF (15 mL) was heated in a microwave reactor at 110 °C for 40 min. After being cooled to room temperature, the reaction mixture was diluted with water and extracted with ethyl acetate. The combined extracts were washed with brine and concentrated under vacuum to give a residue. The residue was purified by flash chromatography (0 – 50% ethyl acetate in petroleum ether) to give compound **1** as a light yellow solid (170 mg, 79% yield). ^{1}H NMR (800 MHz, $CDCl_3$) δ 7.49 (d, J = 3.4 Hz, 1H), 7.27 (dd, J = 7.9, 0.8 Hz, 1H), 7.22 (dd, J = 10.3, 5.4 Hz, 1H), 6.95 (d, J = 7.4 Hz, 1H), 4.03 (s, 3H), 2.63 (s, 3H). HRMS (*m/z*): $[M + H]^+$ calculated for $C_{11}H_{11}O_3^+$, 191.0703; found, 191.0697.

1-(((1-(7-Methoxybenzofuran-2-yl)ethyl)amino)methyl)cyclohexan-1-ol Hydrochloride (O_3LE_6). A mixture of **1** (155 mg, 0.815 mmol), 1-(aminomethyl)cyclohexanol (126 mg, 0.978 mmol) and AcOH (47 mg, 0.815 mmol) in anhydrous THF (20 mL) was stirred at 60 °C for 1 h. $NaHB(AcO)_3$ (345 mg, 1.63 mmol) was added and the resulting mixture was stirred at room temperature overnight. The reaction mixture was diluted with water and extracted with ethyl acetate twice. The combined extracts were washed with brine and concentrated in vacuum to give a residue. The residue was purified by flash chromatography (0 – 5% methanol in dichloromethane) to give a white solid (23 mg, 9% yield). The solid was dissolved in a mixture of methanol and dichloromethane (1:10, 5 mL) and then treated with 4 *M* HCl (g) in dioxane (1 mL). Removal of

volatiles gave the title compound **O_3LE_6** as a white solid (16 mg, 82% yield). HPLC: 95.3%, t_R = 12.4 min; ^{1}H NMR (800 MHz, CD_3OD) δ 7.23 – 7.19 (m, 2H), 7.06 (s, 1H), 6.96 (dd, J = 6.3, 2.6 Hz, 1H), 4.78 (q, J = 7.0 Hz, 1H), 3.98 (s, 3H), 2.92 and 2.88 (ABq, J = 12.7 Hz, 2H), 1.82 (d, J = 7.0 Hz, 3H), 1.66 – 1.57 (m, 4H), 1.54 – 1.39 (m, 5H), 1.33 – 1.27 (m, 1H). ^{13}C NMR (201 MHz, CD_3OD) δ 152.3, 147.0, 146.0, 130.5, 125.5, 114.8, 109.5, 108.8, 69.5, 56.5, 55.3, 53.4, 36.2 (2C), 26.38, 22.6 (2C), 16.1. HRMS (*m/z*): $[M + H]^+$ calculated for $C_{18}H_{26}NO_3^+$, 304.1907; found, 304.1907.

1-(7-Ethoxybenzofuran-2-yl)ethan-1-one (2). A mixture of 2-acetyl-7-hydrobenzofuran (1.0 g, 5.68 mmol), iodoethane (1.33 g, 8.51 mmol) and K_2CO_3 (1.57 g, 11.35 mmol) in DMF (15 mL) was heated in a microwave reactor at 110 °C for 45 min. After being cooled to room temperature, the reaction mixture was diluted with water and extracted with ethyl acetate. The combined extracts were washed with brine and concentrated under vacuum to give a residue. The residue was purified by flash chromatography (0 – 50% ethyl acetate in petroleum ether) to give compound **2** as a white solid (940 mg, 81% yield). ^{1}H NMR (800 MHz, $CDCl_3$) δ 7.48 (s, 1H), 7.26 (dd, J = 7.6, 1.0 Hz, 1H), 7.20 (dd, J = 10.4, 5.4 Hz, 1H), 6.94 (d, J = 7.8 Hz, 1H), 4.28 (q, J = 7.0 Hz, 2H), 2.63 (s, 3H), 1.53 (t, J = 7.0 Hz, 3H). HRMS (*m/z*): $[M + H]^+$ calculated for $C_{12}H_{13}O_3^+$, 205.0859; found, 205.0867.

1-(((1-(7-Ethoxybenzofuran-2-yl)ethyl)amino)methyl)cyclohexan-1-ol Hydrochloride (O_4LE_6). A mixture of **2** (120 mg, 0.588 mmol), 1-(aminomethyl)cyclohexanol (91 mg, 0.705 mmol) and AcOH (35 mg, 0.588 mmol) in anhydrous THF (15 mL) was stirred at 60 °C overnight. $NaHB(AcO)_3$ (249 mg, 1.18 mmol) was added and the resulting mixture was stirred at room temperature for 1 h. The reaction mixture was diluted with water and extracted with ethyl acetate twice. The combined extracts were washed with brine and concentrated in vacuum to give a residue.

The residue was purified by flash chromatography (0 – 5% methanol in dichloromethane) to give a colorless oil (84 mg, 45% yield). The oil was dissolved in a mixture of methanol and dichloromethane (1:10, 5 mL) and then treated with 2 *M* HCl (g) in diethyl ether (2 mL). Removal of volatiles gave the title compound **O_4LE_6** as a white solid (90 mg, 96% yield). HPLC: 99.4%, t_R = 13.0 min; ^{1}H NMR (600 MHz, CD_3OD) δ 7.21 – 7.17 (m, 2H), 7.04 (s, 1H), 6.94 (dd, J = 6.9, 2.0 Hz, 1H), 4.76 (q, J = 7.0 Hz, 1H), 4.24 (q, J = 7.0 Hz, 2H), 2.89 and 2.88 (ABq, J = 12.7 Hz, 2H), 1.81 (d, J = 7.0 Hz, 3H), 1.67 – 1.57 (m, 4H), 1.55 – 1.49 (m, 1H), 1.48 – 1.39 (m, 7H), 1.35 – 1.27 (m, 1H). ^{13}C NMR (201 MHz, CD_3OD) δ 152.2, 146.2 (2C), 130.5, 125.4, 114.7, 109.7, 109.5, 69.5, 65.6, 55.3, 53.4, 36.2 (2C), 26.4, 22.6 (2C), 16.2, 15.2. HRMS (*m/z*): $[M + H]^+$ calculated for $C_{19}H_{28}NO_3^+$, 318.2064; found, 318.2073.

Chiral separation of O_4LE_6:

The racemic **O_4LE_6** was separated by chiral HPLC. Analytical conditions: CHIRALPAK OD-H (ODH0CD-TC013) chiral column (15 cm × 4.6 mm); hexane/isopropanol = 90/10 (V/V) as the fluent phase. Preparative conditions: CHIRALPAK OD-H chiral column (25 cm × 5.0 cm, 10 μM); hexane/ isopropanol = 90/10 (V/V) as the eluent; flow rate = 60 mL/min; λ = 254 nm, 35 °C. Compound **O_4RE_6** was isolated as the first-eluting peaks, with **O_4SE_6** as the second-eluting peaks, both after evaporation appeared as yellow oil. Optical purity of both enantiomers was determined on analysis HPLC after the separation (>98% *ee* for both enantiomers). HCl salts of both enantiomer were obtained using 2 *M* HCl (g) in diethyl ether as described for **O_4LE_6**. Optical rotation values were recorded on Rudolph Autopol VI automatic polarimeter (λ = 589 nm, temperature = 20 °C).

(*R*)-(+)-1-(((1-(7-Ethoxybenzofuran-2-yl)ethyl)amino)methyl)-cyclohexan-1-ol Hydrochloride (O_4RE_6).

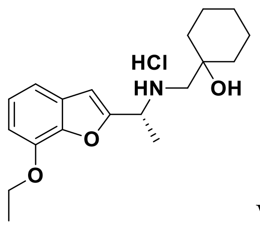

White solid. HPLC: 99.6%, t_R = 13.4 min; ^{1}H NMR (800 MHz, CD_3OD) δ 7.22 – 7.18 (m, 2H), 7.04 (s, 1H), 6.94 (dd, J = 7.2, 1.7 Hz, 1H), 4.77 (q, J = 7.0 Hz, 1H), 4.24 (q, J = 7.0 Hz, 2H), 2.91 and 2.89 (ABq, J = 12.7 Hz, 2H), 1.82 (d, J = 7.0 Hz, 3H), 1.67 – 1.57 (m, 4H), 1.55 – 1.50 (m, 1H), 1.48 – 1.41 (m, 7H), 1.33 – 1.29 (m, 1H). ^{13}C NMR (201 MHz, CD_3OD) δ 152.2, 146.2 (2C), 130.5, 125.4, 114.7, 109.7, 109.5, 69.5, 65.6, 55.3, 53.4, 36.2 (2C), 26.4, 22.6 (2C), 16.2, 15.2. HRMS (*m/z*): [M + H]$^+$ calculated for $C_{19}H_{28}NO_3^+$, 318.2064; found, 318.2060. $[\alpha]_D^{20}$ +0.4 (*c* 1.0, MeOH).

The absolute configuration of **O_4RE_6** was determined by X-ray crystallography of its 4-bromo-benzamide derivative.

4-Bromo-*N*-(1-(7-ethoxybenzofuran-2-yl)ethyl)-*N*-((1-hydroxycyclo-hexyl)methyl)benzamide (O_4RE_6-benzamide). A mixture of **O_4RE_6** (free base, 20 mg, 0.063 mmol), $NaHCO_3$ (11 mg, 0.126 mmol) in dry dioxane (5 mL) was added 4-bromobenzoyl chloride (17 mg, 0.076 mmol). The resulting mixture was stirred at room temperature overnight. The reaction mixture was then concentrated and the residue was purified by flash chromatography (0 – 30% ethyl acetate in petroleum ether) to give the title compound as a white solid (24 mg, 76% yield). ^{1}H NMR (600 MHz, $CDCl_3$) δ 7.59 (d, J = 8.0 Hz, 2H), 7.49 (d, J = 8.0 Hz, 2H), 7.16 – 7.08 (m, 2H), 6.80 (d, J = 7.4 Hz, 1H), 6.53 (s, 1H), 5.17 (q, J = 6.9 Hz, 1H), 4.29 – 4.21 (m, 2H), 3.56 and 3.17 (ABq, J = 14.6 Hz, 2H), 1.74 – 1.55 (m, 8H), 1.52 (t, J = 7.0 Hz, 3H), 1.50 – 1.45 (m, 1H), 1.39 – 1.17 (m, 2H), 1.09 – 0.91 (m, 2H). HRMS (*m/z*): [M + H]$^+$ calculated for $C_{26}H_{31}BrNO_4^+$, 500.1431 and 502.1411; found, 500.1441 and 502.1426. The solid was

recrystallized from diethyl ether to give needle-like crystals that were used for X-ray diffraction.

X-ray Diffraction of O_4RE_6-benzamide. X-ray diffraction of **O_4RE_6-benzamide** crystals was carried out at 220 K on a Bruker Apex II CCD diffractometer using Mo Kα radiation (λ = 0.71073 Å). The structure was solved with the ShelXT structure solution program using Intrinsic Phasing and refined with the ShelXL refinement package using Least Squares minimisation. Crystal data and refinement details are summarized in Supplementary Table 10.

Supplementary Table 10 Crystal data and structure refinement for **O_4RE_6-benzamide**.

formula	$C_{26}H_{30}BrNO_4$
Temperature/K	220
Crystal system	orthorhombic
Space group	$P2_12_12_1$
a (Å)	6.0082(19)
b (Å)	11.550(3)
c (Å)	35.890(3)
α/°	90
β/°	90
γ/°	90
Volume/Å^3	2490.5(10)
Z	4
ρ_{calc}g/cm^3	1.335
μ/mm^{-1}	1.681
F(000)	1040.0
Crystal size/mm^3	0.18 × 0.06 × 0.03
Radiation	MoKα (λ = 0.71073)
2Θ range for data collection/°	3.704 to 50.02
Index ranges	$-7 \le h \le 7$, $-12 \le k \le 13$, $-42 \le l \le 15$
Reflections collected	7485
Independent reflections	3889 [R_{int} = 0.1013, R_{sigma} = 0.1743]
Data/restraints/parameters	3889/255/292
Goodness-of-fit on F^2	1.006
Final R indexes [I>=2σ (I)]	R_1 = 0.0959, wR_2 = 0.2398
Final R indexes [all data]	R_1 = 0.1640, wR_2 = 0.2771
Largest diff. peak/hole / e Å^{-3}	0.91/-0.80
Flack parameter	0.07(2)

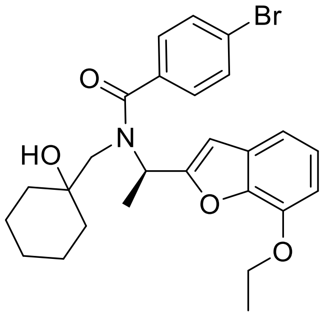

=

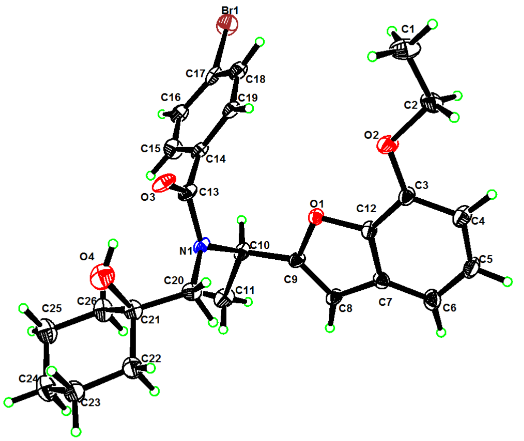

O$_4$RE$_6$-Br

(*S*)-(–)-1-(((1-(7-Ethoxybenzofuran-2-yl)ethyl)amino)methyl)-cyclohexan-1-ol hydrochloride (O$_4$SE$_6$).

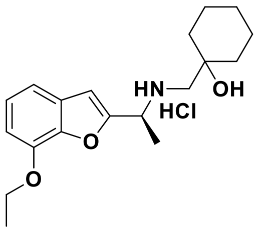

White solid. HPLC: 99.1%, t_R = 13.2 min; ^{1}H NMR (800 MHz, CD_3OD) δ 7.21 – 7.18 (m, 2H), 7.03 (s, 1H), 6.94 (dd, J = 7.1, 1.8 Hz, 1H), 4.75 (q, J = 7.2 Hz, 2H), 4.24 (q, J = 7.0 Hz, 2H), 2.91 – 2.86 (m, 2H), 1.81 (d, J = 6.9 Hz, 3H), 1.68 – 1.57 (m, 4H), 1.55 – 1.50 (m, 1H), 1.48 – 1.41 (m, 7H), 1.33 – 1.28 (m, 1H). ^{13}C NMR (201 MHz, CD_3OD) δ 152.2, 146.2 (2C), 130.5, 125.4, 114.7, 109.7, 109.5, 69.5, 65.6, 55.3, 53.4, 36.2 (2C), 26.4, 22.6 (2C), 16.2, 15.2. HRMS (*m/z*): $[M + H]^+$ calculated for $C_{19}H_{28}NO_3^+$, 318.2064; found, 318.2058. $[\alpha]_D^{20}$ −0.3 (*c* 1.0, MeOH).

1-(7-Propoxybenzofuran-2-yl)ethan-1-one (3). A mixture of 2-acetyl-7-hydrobenzofuran (200 mg, 1.14 mmol), 1-bromopropane (279 mg, 2.27 mmol) and K_2CO_3 (314 mg, 2.27 mmol) in DMF (10 mL) was heated in a microwave reactor at 110 °C for 45 min. After being cooled to room temperature, the reaction mixture was diluted with water and extracted with ethyl acetate. The

combined extracts were washed with brine and concentrated under vacuum to give a residue. The residue was purified by flash chromatography (0 – 50% ethyl acetate in petroleum ether) to give compound **3** as a yellow solid (210 mg, 85% yield). ^{1}H NMR (800 MHz, $CDCl_3$) *δ* 7.48 (d, *J* = 3.5 Hz, 1H), 7.27 – 7.24 (m, 1H), 7.21 – 7.18 (m, 1H), 6.94 (dd, *J* = 7.8, 0.6 Hz, 1H), 4.17 (t, *J* = 6.6 Hz, 2H), 2.63 (s, 3H), 1.97 – 1.87 (m, 2H), 1.10 (t, *J* = 7.4 Hz, 3H). HRMS (*m/z*): $[M + H]^+$ calculated for $C_{13}H_{15}O_3^+$, 219.1016; found, 219.1012.

1-(((1-(7-Propoxybenzofuran-2-yl)ethyl)amino)methyl)cyclohexan-1-ol Hydrochloride (O_5LE_6). A mixture of **3** (120 mg, 0.55 mmol), 1-(aminomethyl)cyclohexanol (85 mg, 0.66 mmol) and AcOH (32 mg, 0.55 mmol) in anhydrous THF (15 mL) was stirred at 60 °C for 12 h. $NaHB(AcO)_3$ (349 mg, 1.65 mmol) was added and the resulting mixture was stirred at 60 °C overnight. Methanol (5 mL) was added and the reaction mixture was stirred for 10 min. The reaction mixture was then concentrated and the residue was purified by flash chromatography (0 – 5% methanol in dichloromethane) to give a colorless oil (53 mg, 29% yield). The oil was dissolved in a mixture of methanol and dichloromethane (1:10, 5 mL) and then treated with 2 *M* HCl (g) in diethyl ether (2 mL). Removal of volatiles gave the title compound **O_5LE_6** as a light yellow solid (52 mg, 88% yield). HPLC: 99.9%, t_R = 17.6 min; ^{1}H NMR (800 MHz, CD_3OD) *δ* 7.23 – 7.15 (m, 2H), 7.05 (s, 1H), 6.94 (dd, *J* = 7.4, 1.5 Hz, 1H), 4.78 (q, *J* = 6.9 Hz, 1H), 4.14 (t, *J* = 6.4 Hz, 2H), 2.92 and 2.89 (ABq, *J* = 12.6 Hz, 2H), 1.89 – 1.84 (m, 2H), 1.82 (d, *J* = 7.0 Hz, 3H), 1.66 – 1.57 (m, 4H), 1.54 – 1.38 (m, 5H), 1.32 – 1.27 (m, 1H), 1.09 (t, *J* = 7.5 Hz, 3H). ^{13}C NMR (201 MHz, CD_3OD) *δ* 152.2, 146.4, 146.2, 130.5, 125.4, 114.7, 109.8, 109.5, 71.6, 69.5, 55.2, 53.4, 36.2 (2C), 26.4, 23.7, 22.6 (2C), 16.1, 10.9. HRMS (*m/z*): $[M + H]^+$ calculated for $C_{20}H_{30}NO_3^+$, 332.2220; found, 332.2211.

1-(7-Butoxybenzofuran-2-yl)ethan-1-one (4). A mixture of 2-acetyl-7-hydrobenzofuran (168 mg,

0.95 mmol), 1-bromobutane (196 mg, 1.4 mmol) and K_2CO_3 (395 mg, 2.9 mmol) in acetonitrile (20 mL) was stirred at 80 °C overnight. After being cooled to room temperature, the reaction mixture was diluted with water and extracted with ethyl acetate. The combined extracts were washed with brine and concentrated under vacuum to give a residue. The residue was purified by flash chromatography (0 – 30% ethyl acetate in petroleum ether) to give compound **4** as a light yellow solid (193 mg, 87% yield). ^{1}H NMR (800 MHz, $CDCl_3$) δ 7.48 (s, 1H), 7.26 – 7.25 (m, 1H), 7.20 (t, J = 7.8 Hz, 1H), 6.94 (d, J = 7.5 Hz, 1H), 4.21 (t, J = 6.6 Hz, 2H), 2.63 (s, 3H), 1.91 – 1.85 (m, 2H), 1.58 – 1.54 (m, 2H), 1.01 (t, J = 7.4 Hz, 3H). HRMS (*m/z*): [M + H]$^+$ calculated for $C_{14}H_{17}O_3^+$, 233.1172; found, 233.1163.

1-(((1-(7-Butoxybenzofuran-2-yl)ethyl)amino)methyl)cyclohexan-1-ol Hydrochloride (O_6LE_6). A mixture of **4** (170 mg, 0.732 mmol), 1-(aminomethyl)cyclohexanol (94 mg, 0.732 mmol) and AcOH (44 mg, 0.732 mmol) in anhydrous THF (15 mL) was stirred at 60 °C overnight. $NaHB(AcO)_3$ (310 mg, 1.46 mmol) was added and the resulting mixture was stirred at 60 °C for 1 h. Methanol (5 mL) was added and the reaction mixture was stirred for 1 h. The reaction mixture was then concentrated and the residue was purified by flash chromatography (0 – 5% methanol in dichloromethane) to give a yellow oil (110 mg, 44% yield). The oil was dissolved in a mixture of methanol and dichloromethane (1:10, 5 mL) and then treated with 2 *M* HCl (g) in diethyl ether (2 mL). Removal of volatiles gave the title compound **O_6LE_6** as a yellow solid (114 mg, 94% yield). HPLC: 98.0%, t_R = 17.5 min; ^{1}H NMR (800 MHz, CD_3OD) δ 7.21 – 7.17 (m, 2H), 7.05 (s, 1H), 6.94 (dd, J = 7.4, 1.5 Hz, 1H), 4.78 (q, J = 6.9 Hz, 1H), 4.18 (t, J = 6.3 Hz, 2H), 2.92 and 2.89 (ABq, J = 12.7 Hz, 2H), 1.85 – 1.79 (m, 5H), 1.66 – 1.58 (m, 4H), 1.58 – 1.54 (m, 2H), 1.54 – 1.39 (m, 5H), 1.32 – 1.27 (m, 1H), 1.01 (t, J = 7.4 Hz, 3H). ^{13}C NMR (201 MHz, CD_3OD) δ 152.2, 146.4, 146.2, 130.5, 125.4, 114.7, 109.7, 109.5, 69.7, 69.5, 55.2, 53.4, 36.2 (2C), 32.6, 26.4, 22.6

(2C), 20.3, 16.2, 14.2. HRMS (*m/z*): $[M + H]^+$ calculated for $C_{21}H_{32}NO_3^+$, 346.2377; found, 346.2381.

Synthesis of compounds $O_4LE_{1\sim5}$ and $O_4LE_{7\sim9}$:

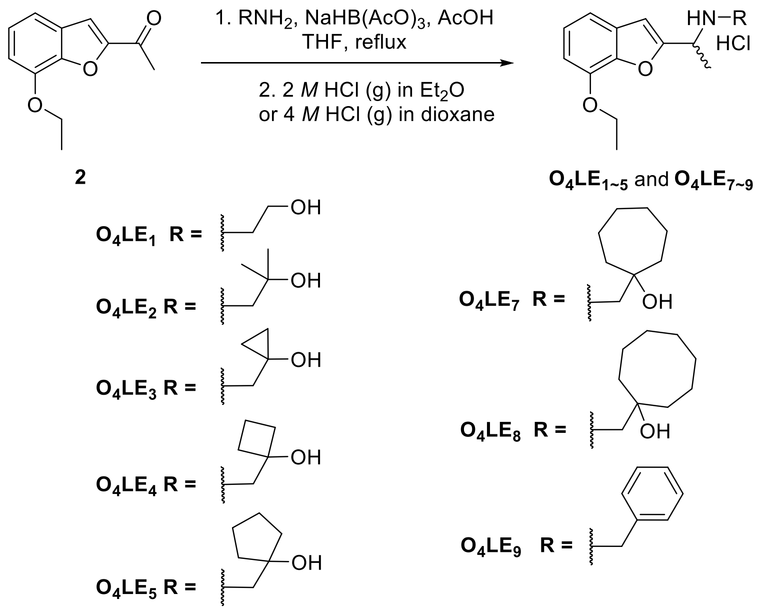

2-((1-(7-Ethoxybenzofuran-2-yl)ethyl)amino)ethan-1-ol Hydrochloride (O_4LE_1). A mixture of **2** (93 mg, 0.455 mmol), ethanolamine (66 mg, 0.911 mmol) and AcOH (27 mg, 0.455 mmol) in anhydrous THF (15 mL) was stirred at 60 °C overnight. $NaHB(AcO)_3$ (289 mg, 1.37 mmol) was added and the resulting mixture was stirred at room temperature for 1 h. The reaction mixture was diluted with water and extracted with ethyl acetate twice. The combined extracts were washed with brine and concentrated in vacuum to give a residue. The residue was purified by flash chromatography (0 – 5% methanol in dichloromethane) to give a colorless oil (35 mg, 31% yield). The oil was dissolved in a mixture of methanol and dichloromethane (1:10, 5 mL) and then treated with 4 *M* HCl (g) in dioxane (1 mL). Removal of volatiles gave the title compound **O_4LE_1** as a light yellow oil (40 mg, 99% yield). HPLC: 98.7%, t_R = 11.1 min; ^1H NMR (800 MHz, CD_3OD) δ 7.21 – 7.16 (m, 2H), 7.04 (s, 1H), 6.92 (dd, J = 7.6, 1.2 Hz, 1H), 4.79 (q, J = 6.9 Hz, 1H), 4.23

(q, J = 7.0 Hz, 2H), 3.83 – 3.75 (m, 2H), 3.12 – 3.08 (m, 1H), 3.05 – 3.00 (m, 1H), 1.81 (d, J = 7.0 Hz, 3H), 1.45 (t, J = 7.0 Hz, 3H). ^{13}C NMR (201 MHz, CD_3OD) δ 152.3, 146.1 (2C), 130.5, 125.4, 114.7, 109.7, 109.2, 65.5, 57.9, 52.7, 48.5, 16.7, 15.2. HRMS (*m/z*): $[M + H]^+$ calculated for $C_{14}H_{20}NO_3^+$, 250.1438; found, 250.1428.

1-((1-(7-Ethoxybenzofuran-2-yl)ethyl)amino)-2-methylpropan-2-ol Hydrochloride (O_4LE_2).

A mixture of **2** (110 mg, 0.539 mmol), 1,1-dimethylethanolamine (58 mg, 0.646 mmol) and AcOH (33 mg, 0.539 mmol) in anhydrous THF (15 mL) was stirred at 60 °C overnight. $NaHB(AcO)_3$ (228 mg, 1.08 mmol) was added and the resulting mixture was stirred at room temperature for 1 h. The reaction mixture was diluted with water and extracted with ethyl acetate twice. The combined extracts were washed with brine and concentrated in vacuum to give a residue. The residue was purified by flash chromatography (0 – 5% methanol in dichloromethane) to give a colorless oil (18 mg, 12% yield). The oil was dissolved in a mixture of methanol and dichloromethane (1:10, 5 mL) and then treated with 2 *M* HCl (g) in diethyl ether (2 mL). Removal of volatiles gave the title compound **O_4LE_2** as a yellow solid (17 mg, 83% yield). HPLC: 97.8%, t_R = 12.2 min; ^{1}H NMR (800 MHz, CD_3OD) δ 7.22 – 7.17 (m, 2H), 7.05 (s, 1H), 6.93 (dd, J = 7.6, 1.4 Hz, 1H), 4.79 (q, J = 6.9 Hz, 1H), 4.23 (q, J = 7.0 Hz, 2H), 2.92 and 2.89 (ABq, J = 12.5 Hz, 2H), 1.82 (d, J = 7.0 Hz, 3H), 1.45 (t, J = 7.0 Hz, 3H), 1.27 (s, 3H), 1.25 (s, 3H). ^{13}C NMR (201 MHz, CD_3OD) δ 152.1, 146.2 (2C), 130.5, 125.4, 114.7, 109.8, 109.5, 68.3, 65.6, 56.0, 53.5, 27.6 (2C), 16.2, 15.2. HRMS (*m/z*): $[M + H]^+$ calculated for $C_{16}H_{24}NO_3^+$, 278.1751; found, 278.1753.

1-(((1-(7-Ethoxybenzofuran-2-yl)ethyl)amino)methyl)cyclopropan-1-ol Hydrochloride (O_4LE_3). A mixture of **2** (140 mg, 0.686 mmol), 1-(aminomethyl)cyclopropanol (72 mg, 0.823 mmol) and AcOH (82 mg, 1.37 mmol) in anhydrous THF (15 mL) was stirred at 60 °C for 24 h. $NaHB(AcO)_3$ (290 mg, 1.37 mmol) was added and the resulting mixture was stirred at 60 °C for 2

h. Methanol (5 mL) was added and the reaction mixture was stirred for 10 min. The reaction mixture was then concentrated and the residue was purified by flash chromatography (0 – 5% methanol in dichloromethane) to give a colorless oil (21 mg, 11% yield). The oil was dissolved in a mixture of methanol and dichloromethane (1:10, 5 mL) and then treated with 4 *M* HCl (g) in dioxane (1 mL). Removal of volatiles gave the title compound **O_4LE_3** as a yellow solid (23 mg, 97% yield). HPLC: 96.6%, t_R = 12.3 min; ^1H NMR (800 MHz, CD_3OD) δ 7.21 – 7.16 (m, 2H), 7.03 (s, 1H), 6.93 (dd, *J* = 7.4, 1.5 Hz, 1H), 4.83 (q, *J* = 7.0 Hz, 1H), 4.23 (q, *J* = 7.0 Hz, 2H), 3.08 and 3.04 (ABq, *J* = 13.2 Hz, 2H), 1.82 (d, *J* = 6.9 Hz, 3H), 1.45 (t, *J* = 7.0 Hz, 3H), 0.89 – 0.83 (m, 2H), 0.73 – 0.70 (m, 1H), 0.67 – 0.63 (m, 1H). ^{13}C NMR (201 MHz, CD_3OD) δ 152.4, 146.1 (2C), 130.5, 125.4, 114.7, 109.67, 109.2, 65.5, 54.0, 52.67, 51.9, 16.7, 15.2, 13.2, 13.1. HRMS (*m/z*): $[M + H]^+$ calculated for $C_{16}H_{22}NO_3^+$, 276.1594; found, 276.1597.

1-(((1-(7-Ethoxybenzofuran-2-yl)ethyl)amino)methyl)cyclobutan-1-ol Hydrochloride (O_4LE_4). A mixture of **2** (114 mg, 0.558 mmol), 1-(aminomethyl)cyclobutanol (68 mg, 0.670 mmol) and AcOH (67 mg, 1.12 mmol) in anhydrous THF (15 mL) was stirred at 60 °C for 10 h. $NaHB(AcO)_3$ (237 mg, 1.12 mmol) was added and the resulting mixture was stirred at 60 °C for 8 h. Methanol (5 mL) was added and the reaction mixture was stirred for 10 min. The reaction mixture was then concentrated and the residue was purified by flash chromatography (0 – 5% methanol in dichloromethane) to give a colorless oil (31 mg, 19% yield). The oil was dissolved in a mixture of methanol and dichloromethane (1:10, 5 mL) and then treated with 4 *M* HCl (g) in dioxane (1 mL). Removal of volatiles gave the title compound **O_4LE_4** as a brown solid (34 mg, 97% yield). HPLC: 99.5%, t_R = 12.9 min; ^1H NMR (800 MHz, CD_3OD) δ 7.22 – 7.18 (m, 2H), 7.04 (s, 1H), 6.95 (dd, *J* = 7.2, 1.6 Hz, 1H), 4.77 (q, *J* = 7.0 Hz, 1H), 4.25 (q, *J* = 7.0 Hz, 2H), 3.11 and 3.07 (ABq, *J* = 12.7 Hz, 2H), 2.17 – 2.06 (m, 4H), 1.81 (d, *J* = 7.0 Hz, 3H), 1.76 – 1.70 (m,

1H), 1.51 – 1.48 (m, 1H), 1.46 (q, $J = 7.3$ Hz, 3H). ^{13}C NMR (201 MHz, CD_3OD) δ 152.3, 146.2, 146.1, 130.5, 125.4, 114.7, 109.8, 109.3, 72.4, 65.6, 53.2, 52.9, 35.2, 35.1, 16.4, 15.2, 12.2. HRMS (*m/z*): $[M + H]^+$ calculated for $C_{17}H_{24}NO_3^+$, 290.1751; found, 290.1756.

1-(((1-(7-Ethoxybenzofuran-2-yl)ethyl)amino)methyl)cyclopentan-1-ol Hydrochloride (O_4LE_5). A mixture of **2** (160 mg, 0.784 mmol), 1-(aminomethyl)cyclopentanol (108 mg, 0.940 mmol) and AcOH (95 mg, 1.57 mmol) in anhydrous THF (15 mL) was stirred at 60 °C overnight. $NaHB(AcO)_3$ (332 mg, 1.57 mmol) was added and the resulting mixture was stirred at 60 °C for 8 h. Methanol (5 mL) was added and the reaction mixture was stirred for 10 min. The reaction mixture was then concentrated and the residue was purified by flash chromatography (0 – 5% methanol in dichloromethane) to give a colorless oil (29 mg, 12% yield). The oil was dissolved in a mixture of methanol and dichloromethane (1:10, 5 mL) and then treated with 4 *M* HCl (g) in dioxane (1 mL). Removal of volatiles gave the title compound **O_4LE_5** as a yellow solid (31 mg, 95% yield). HPLC: 99.0%, $t_R = 13.1$ min; ^1H NMR (800 MHz, CD_3OD) δ 7.21 – 7.17 (m, 2H), 7.05 (s, 1H), 6.93 (dd, $J = 7.6, 1.4$ Hz, 1H), 4.80 (q, $J = 7.0$ Hz, 1H), 4.23 (q, $J = 7.0$ Hz, 2H), 3.04 and 3.01 (ABq, $J = 12.6$ Hz, 2H), 1.85 – 1.79 (m, 5H), 1.77 – 1.72 (m, 2H), 1.66 – 1.55 (m, 4H), 1.45 (t, $J = 7.0$ Hz, 3H). ^{13}C NMR (201 MHz, CD_3OD) δ 152.2, 146.2, 146.1, 130.5, 125.4, 114.7, 109.7, 109.5, 79.4, 65.6, 54.8, 53.4, 38.9, 38.8, 24.6, 24.5, 16.3, 15.2. HRMS (*m/z*): $[M + H]^+$ calculated for $C_{18}H_{26}NO_3^+$, 304.1907; found, 304.1916.

1-(((1-(7-Ethoxybenzofuran-2-yl)ethyl)amino)methyl)cycloheptan-1-ol Hydrochloride (O_4LE_7). A mixture of **2** (140 mg, 0.686 mmol), 1-(aminomethyl)cycloheptanol (118 mg, 0.823 mmol) and AcOH (82 mg, 1.37 mmol) in anhydrous THF (20 mL) was stirred at 60 °C overnight. $NaHB(AcO)_3$ (290 mg, 1.37 mmol) was added and the resulting mixture was stirred at 60 °C for 8 h. Methanol (5 mL) was added and the reaction mixture was stirred for 10 min. The reaction

mixture was then concentrated and the residue was purified by flash chromatography (0 – 5% methanol in dichloromethane) to give a colorless oil (30 mg, 13% yield). The oil was dissolved in a mixture of methanol and dichloromethane (1:10, 5 mL) and then treated with 2 *M* HCl (g) in diethyl ether (2 mL). Removal of volatiles gave the title compound **O_4LE_7** as a yellow solid (29 mg, 87% yield). HPLC: 99.0%, t_R = 14.4 min; ^{1}H NMR (800 MHz, CD_3OD) δ 7.21 – 7.17 (m, 2H), 7.05 – 7.03 (m, 1H), 6.94 (dt, *J* = 7.5, 1.7 Hz, 1H), 4.77 (q, *J* = 6.4 Hz, 1H), 4.24 (q, *J* = 7.0 Hz, 2H), 2.90 and 2.87 (ABq, *J* = 12.7 Hz, 2H), 1.81 (d, *J* = 7.0 Hz, 3H), 1.73 – 1.56 (m, 8H), 1.52 – 1.47 (m, 2H), 1.46 (t, *J* = 7.0 Hz, 3H), 1.39 – 1.30 (m, 2H). ^{13}C NMR (201 MHz, CD_3OD) δ 152.2, 146.2 (2C), 130.5, 125.4, 114.7, 109.7, 109.5, 73.5, 65.6, 55.2, 53.5, 39.7 (2C), 30.9 (2C), 23.0, 22.9, 16.2, 15.2. HRMS (*m/z*): $[M + H]^+$ calculated for $C_{20}H_{30}NO_3^+$, 332.2220; found, 332.2221.

1-(((1-(7-Ethoxybenzofuran-2-yl)ethyl)amino)methyl)cyclooctan-1-ol Hydrochloride (O_4LE_8). A mixture of **2** (100 mg, 0.49 mmol), 1-(aminomethyl)cyclooctanol (92 mg, 0.59 mmol) and AcOH (29 mg, 0.49 mmol) in anhydrous THF (10 mL) was stirred at 60 °C for 4 h. $NaHB(AcO)_3$ (208 mg, 0.98 mmol) was added and the resulting mixture was stirred at 60 °C overnight. The reaction mixture was diluted with water and extracted with ethyl acetate. The combined extracts were washed with brine and concentrated in vacuum to give a residue. The residue was purified by flash chromatography (0 – 3% methanol in dichloromethane) to give a yellow oil (43 mg, 25% yield). The oil was dissolved in a mixture of methanol and dichloromethane (1:10, 5 mL) and then treated with 2 *M* HCl (g) in diethyl ether (2 mL). Removal of volatiles gave the title compound **O_4LE_8** as a yellow solid (45 mg, 95% yield). HPLC: 98.1%, t_R = 18.0 min; ^{1}H NMR (800 MHz, CD_3OD) δ 7.22 – 7.17 (m, 2H), 7.05 (s, 1H), 6.94 (d, *J* = 7.4 Hz, 1H), 4.78 (q, *J* = 6.9 Hz, 1H), 4.24 (q, *J* = 7.0 Hz, 2H), 2.90 and 2.89 (ABq, *J* = 12.8 Hz, 2H),

1.82 (d, J = 7.0 Hz, 3H), 1.79 – 1.75 (m, 2H), 1.65 – 1.58 (m, 8H), 1.50 – 1.38 (m, 7H). ^{13}C NMR (201 MHz, CD_3OD) δ 150.8, 144.8, 129.1, 124.1, 113.3, 108.4, 108.1, 71.6, 66.7, 64.2, 52.7, 52.1, 33.4, 33.3, 27.7, 27.6, 24.5, 21.4, 21.3, 14.7, 13.8. HRMS (*m/z*): $[M + H]^+$ calculated for $C_{21}H_{32}NO_3^+$, 346.2377; found, 346.2376.

***N*-Benzyl-1-(7-ethoxybenzofuran-2-yl)ethan-1-amine Hydrochloride (O_4LE_9).** A mixture of **2** (105 mg, 0.514 mmol), phenylmethanamine (110 mg, 1.03 mmol) and AcOH (30 mg, 0.514 mmol) in anhydrous THF (15 mL) was stirred at 60 °C overnight. $NaHB(AcO)_3$ (326 mg, 1.54 mmol) was added and the resulting mixture was stirred at room temperature for 1 h. The reaction mixture was diluted with water and extracted with ethyl acetate twice. The combined extracts were washed with brine and concentrated in vacuum to give a residue. The residue was purified by flash chromatography (0 – 5% methanol in dichloromethane) to give a colorless oil (95 mg, 63% yield). A part of the oil (30 mg) was dissolved in a mixture of methanol and dichloromethane (1:10, 5 mL) and then treated with 4 M HCl (g) in dioxane (1 mL). Removal of volatiles gave the title compound **O_4LE_9** as a white solid (28 mg, 83% yield). HPLC: 98.5%, t_R = 13.7 min; ^{1}H NMR (800 MHz, CD_3OD) δ 7.47 – 7.41 (m, 5H), 7.23 – 7.18 (m, 2H), 7.07 (s, 1H), 6.95 (dd, J = 7.6, 1.3 Hz, 1H), 4.79 (q, J = 6.9 Hz, 1H), 4.25 (q, J = 7.0 Hz, 2H), 4.20 and 4.08 (ABq, J = 13.2 Hz, 2H), 1.83 (d, J = 7.0 Hz, 3H), 1.47 (t, J = 7.0 Hz, 3H). ^{13}C NMR (201 MHz, CD_3OD) δ 152.2, 146.2, 146.1, 132.3, 131.1 (2C), 130.6, 130.5, 130.2 (2C), 125.5, 114.7, 109.7, 109.3, 65.5, 52.8, 50.4, 17.0, 15.2. HRMS (*m/z*): $[M + H]^+$ calculated for $C_{19}H_{22}NO_2^+$, 296.1645; found, 296.1639.

Synthesis of compounds O_7LE_6 and O_8LE_6:

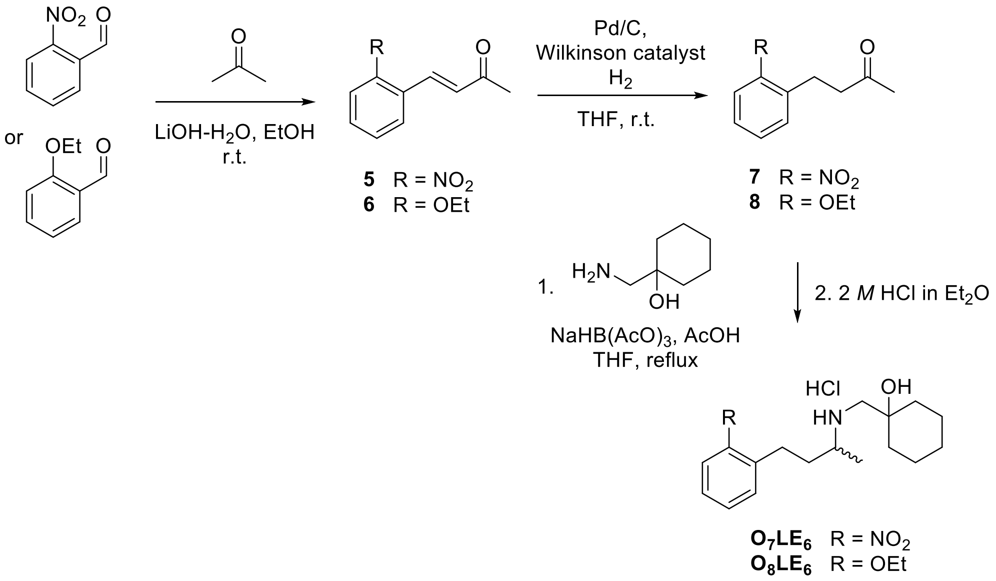

(*E*)-4-(2-Nitrophenyl)but-3-en-2-one (5). A mixture of 2-nitrobenzaldehyde (2.0 g, 13.2 mmol), acetone (9.6 mL, 132 mmol) and lithium hydroxide monohydrate (56 mg, 1.32 mmol) in ethanol (20 mL) was stirred at room temperature for 4 h. The reaction mixture was diluted with water and extracted with ethyl acetate. The combined extracts were washed with brine and concentrated in vacuum to give a residue. The residue was purified by flash chromatography (0 – 30% ethyl acetate in petroleum ether) to give compound **5** as a yellow oil (574 mg, 23% yield). ^{1}H NMR (800 MHz, $CDCl_3$) δ 8.08 (d, J = 8.2 Hz, 1H), 7.98 (d, J = 16.2 Hz, 1H), 7.69 – 7.64 (m, 2H), 7.59 – 7.54 (m, 1H), 6.57 (d, J = 16.2 Hz, 1H), 2.43 (s, 3H). HRMS (*m/z*): $[M + H]^+$ calculated for $C_{10}H_{10}NO_3^+$, 192.0655; found, 192.0657.

4-(2-Nitrophenyl)butan-2-one (7). A mixture of compound **5** (230 mg, 1.2 mmol), Wilkinson catalyst (111 mg, 0.12 mmol) and 10% Pd/C (23 mg) in tetrahydrofuran (15 mL) was stirred at room temperature for 2 h under H_2 atmosphere. After filtration, the filtrate was concentrated in vacuum to give a brown residue. The residue was purified by flash chromatography (0 – 15% ethyl acetate in petroleum ether) to give compound **7** as a yellow oil (63 mg, 27% yield). ^{1}H NMR (800 MHz, $CDCl_3$) δ 7.93 (d, J = 8.1 Hz, 1H), 7.52 (t, J = 7.5 Hz, 1H), 7.40 (d, J = 7.5 Hz, 1H), 7.36 (t,

J = 7.7 Hz, 1H), 3.14 (t, J = 7.5 Hz, 2H), 2.85 (t, J = 7.5 Hz, 2H), 2.16 (s, 3H). HRMS (*m/z*): $[M + H]^+$ calculated for $C_{10}H_{12}NO_3^+$, 194.0812; found, 194.0814.

1-(((4-(2-Nitrophenyl)butan-2-yl)amino)methyl)cyclohexan-1-ol Hydrochloride (O_7LE_6). A mixture of **7** (130 mg, 0.673 mmol), 1-(aminomethyl)cyclohexanol (87 mg, 0.673 mmol) and AcOH (40 mg, 0.673 mmol) in anhydrous dichloromethane (5 mL) and acetonitrile (5 mL) was stirred at 60 °C overnight. $NaHB(AcO)_3$ (285 mg, 1.35 mmol) was added and the resulting mixture was stirred at room temperature for 1 h. Methanol (5 mL) was added and the reaction mixture was stirred for 30 min. The reaction mixture was then concentrated and the residue was purified by flash chromatography (0 – 6% methanol in dichloromethane) to give a yellow oil (130 mg, 63% yield). A part of the oil (100 mg) was dissolved in a mixture of methanol and dichloromethane (1:10, 5 mL) and then treated with 2 *M* HCl (g) in diethyl ether (2 mL). Removal of volatiles gave the title compound $\mathbf{O_7LE_6}$ as a white solid (107 mg, 96% yield). HPLC: 99.1%, t_R = 12.4 min; ^1H NMR (800 MHz, CD_3OD) δ 7.97 (dd, J = 8.1, 0.8 Hz, 1H), 7.65 (td, J = 7.6, 1.0 Hz, 1H), 7.54 (d, J = 7.7 Hz, 1H), 7.49 – 7.46 (m, 1H), 3.41 – 3.36 (m, 1H), 3.07 – 3.02 (m, 1H),3.00 and 2.98 (ABq, J = 12.7 Hz, 2H), 2.93 – 2.89 (m, 1H), 2.18 – 2.13 (m, 1H), 1.97 – 1.91 (m, 1H), 1.71 – 1.66 (m, 2H), 1.66 – 1.62 (m, 2H), 1.59 – 1.48 (m, 5H), 1.45 (d, J = 6.6 Hz, 3H), 1.41 – 1.35 (m, 1H). ^{13}C NMR (201 MHz, CD_3OD) δ 150.6, 136.7, 134.6, 133.4, 129.0, 125.89, 69.5, 56.3, 54.3, 36.3 (2C), 34.7, 30.2, 26.5, 22.6 (2C), 16.1. HRMS (*m/z*): $[M + H]^+$ calculated for $C_{17}H_{27}N_2O_3^+$, 307.2016; found, 307.2026.

(*E*)-4-(2-Ethoxyphenyl)but-3-en-2-one (6). A mixture of 2-ethoxybenzaldehyde (3.0 g, 19.98 mmol), acetone (3.48 g, 59.93 mmol) and lithium hydroxide monohydrate (167 mg, 4.0 mmol) in ethanol (20 mL) was stirred at room temperature overnight. The reaction mixture was diluted with water and extracted with ethyl acetate. The combined extracts were washed with brine and

concentrated in vacuum to give a residue. The residue was purified by flash chromatography (0 – 30% ethyl acetate in petroleum ether) to give compound **6** as a yellow oil (1.19 g, 31% yield). ^{1}H NMR (800 MHz, $CDCl_3$) δ 7.90 (d, J = 16.5 Hz, 1H), 7.54 (d, J = 7.7 Hz, 1H), 7.34 (t, J = 7.8 Hz, 1H), 6.95 (t, J = 7.5 Hz, 1H), 6.90 (d, J = 8.3 Hz, 1H), 6.77 (d, J = 16.5 Hz, 1H), 4.11 (q, J = 7.0 Hz, 2H), 2.38 (s, 3H), 1.48 (t, J = 7.0 Hz, 3H). HRMS (*m/z*): $[M + H]^+$ calculated for $C_{12}H_{15}O_2^+$, 191.1067; found, 191.1068.

4-(2-Ethoxyphenyl)butan-2-one (8). A mixture of compound **6** (1.19 g, 6.26 mmol) and 10% Pd/C (120 mg) in tetrahydrofuran (35 mL) was stirred at room temperature for 1 h under H_2 atmosphere. After filtration, the filtrate was concentrated in vacuum to give a residue. The residue was purified by flash chromatography (0 – 20% ethyl acetate in petroleum ether) to give compound **8** as a colorless oil (900 mg, 75% yield). ^{1}H NMR (600 MHz, $CDCl_3$) δ 7.16 (t, J = 7.8 Hz, 1H), 7.13 (d, J = 7.3 Hz, 1H), 6.86 (t, J = 7.4 Hz, 1H), 6.82 (d, J = 8.1 Hz, 1H), 4.04 (q, J = 6.9 Hz, 2H), 2.92 – 2.85 (m, 2H), 2.78 – 2.67 (m, 2H), 2.14 (s, 3H), 1.42 (t, J = 7.0 Hz, 3H). HRMS (*m/z*): $[M + H]^+$ calculated for $C_{12}H_{17}O_2^+$, 193.1223; found, 193.1224.

1-(((4-(2-ethoxyphenyl)butan-2-yl)amino)methyl)cyclohexan-1-ol Hydrochloride (O_8LE_6). A mixture of **8** (150 mg, 0.78 mmol), 1-(aminomethyl)cyclohexanol (100 mg, 0.78 mmol) and AcOH (47 mg, 0.78 mmol) in anhydrous acetonitrile (10 mL) was stirred at 60 °C overnight. $NaHB(AcO)_3$ (330 mg, 1.56 mmol) was added and the resulting mixture was stirred at 60 °C for 1 h. Methanol (5 mL) was added and the reaction mixture was stirred for 30 min. The reaction mixture was then concentrated and the residue was purified by flash chromatography (0 – 6% methanol in dichloromethane) to give a light yellow oil (146 mg, 61% yield). A part of the oil (126 mg) was dissolved in a mixture of methanol and dichloromethane (1:10, 5 mL) and then treated with 2 *M* HCl (g) in diethyl ether (2 mL). Removal of volatiles gave the title compound **O_8LE_6** as

a white solid (138 mg, 98% yield). HPLC: 96.5% (λ = 280 nm), t_R = 13.1 min; ^{1}H NMR (600 MHz, CD_3OD) δ 7.20 – 7.15 (m, 2H), 6.93 (d, J = 8.0 Hz, 1H), 6.87 (td, J = 7.4, 0.9 Hz, 1H), 4.07 (q, J = 7.0 Hz, 2H), 3.27 – 3.20 (m, 1H), 2.95 – 2.89 (m, 2H), 2.82 – 2.76 (m, 1H), 2.71 – 2.62 (m, 1H), 2.13 – 2.06 (m, 1H), 1.89 – 1.79 (m, 1H), 1.71 – 1.60 (m, 4H), 1.59 – 1.45 (m, 5H), 1.45 – 1.39 (m, 6H), 1.38 – 1.29 (m, 1H). ^{13}C NMR (201 MHz, CD_3OD) δ 158.1, 131.1, 129.8, 128.9, 121.7, 112.6, 69.4, 64.7, 56.1, 54.2, 36.3 (2C), 34.0, 27.5, 26.5, 22.6 (2C), 16.2, 15.3. HRMS (*m/z*): $[M + H]^+$ calculated for $C_{19}H_{32}NO_2^+$, 306.2428; found, 306.2436.

References

1 Lyu, J. *et al.* Ultra-large library docking for discovering new chemotypes. *Nature* **566**, 224-229 (2019).
