# Supplementary material for: Haloperidol bound D2 dopamine receptor structure inspired the discovery of subtype selective ligands
Source: Nat Commun. 2020 Feb 26;11:1074. doi: 10.1038/s41467-020-14884-y (PMC7044277; doi:10.1038/s41467-020-14884-y)
Supplement: Supplementary file 3 — Reporting Summary [file 41467_2020_14884_MOESM3_ESM.pdf]

nature research

Corresponding author(s): Sheng Wang; Jianjun Cheng

Last updated by author(s): Dec 12, 2019

Reporting Summary

Nature Research wishes to improve the reproducibility of the work that we publish. This form provides structure for consistency and transparency in reporting. For further information on Nature Research policies, see Authors & Referees and the Editorial Policy Checklist .

Statistics

For all statistical analyses, confirm that the following items are present in the figure legend, table legend, main text, or Methods section.

n/a	Confirmed	
[ ]	[x]	The exact sample size (*n*) for each experimental group/condition, given as a discrete number and unit of measurement
[ ]	[x]	A statement on whether measurements were taken from distinct samples or whether the same sample was measured repeatedly
[ ]	[x]	The statistical test(s) used AND whether they are one- or two-sided<br>*Only common tests should be described solely by name; describe more complex techniques in the Methods section.*
[x]	[ ]	A description of all covariates tested
[ ]	[x]	A description of any assumptions or corrections, such as tests of normality and adjustment for multiple comparisons
[ ]	[x]	A full description of the statistical parameters including central tendency (e.g. means) or other basic estimates (e.g. regression coefficient) AND variation (e.g. standard deviation) or associated estimates of uncertainty (e.g. confidence intervals)
[ ]	[x]	For null hypothesis testing, the test statistic (e.g. *F*, *t*, *r*) with confidence intervals, effect sizes, degrees of freedom and *P* value noted<br>*Give P values as exact values whenever suitable.*
[x]	[ ]	For Bayesian analysis, information on the choice of priors and Markov chain Monte Carlo settings
[x]	[ ]	For hierarchical and complex designs, identification of the appropriate level for tests and full reporting of outcomes
[x]	[ ]	Estimates of effect sizes (e.g. Cohen's *d*, Pearson's *r*), indicating how they were calculated

Our web collection on statistics for biologists contains articles on many of the points above.

Software and code

Policy information about availability of computer code

Data collection	No custom code was used for data collection. All commercial code used for data collection is stated in the methods
Data analysis	No custom code was used for data analysis. All commercial code used for data analysis is stated in the methods.

For manuscripts utilizing custom algorithms or software that are central to the research but not yet described in published literature, software must be made available to editors/reviewers. We strongly encourage code deposition in a community repository (e.g. GitHub). See the Nature Research guidelines for submitting code & software for further information.

Data

Policy information about availability of data

All manuscripts must include a data availability statement. This statement should provide the following information, where applicable:

- Accession codes, unique identifiers, or web links for publicly available datasets
- A list of figures that have associated raw data
- A description of any restrictions on data availability

Macromolecular structure data will be available via the worldwide protein data bank (wwPDB). Generated and analyzed data sets that support the findings of this study are available as source data and from the corresponding authors upon reasonable request.

Field-specific reporting

Please select the one below that is the best fit for your research. If you are not sure, read the appropriate sections before making your selection.

[x] Life sciences [ ] Behavioural & social sciences [ ] Ecological, evolutionary & environmental sciences

Life sciences study design

All studies must disclose on these points even when the disclosure is negative.

Sample size	Number of technical replicates and biological replicates are reported in the figure legends. Sample size was determined based on variability of the response deviating from the mean as indicated by the standard error of the mean (SEM), which is also represented in the figures. Typically, at least three biological replicates were performed so that the SEM was within at least 20% of the mean, but exact number of replicates are indicated per result in figure legends.<br>To process the diffraction data, number of crystals and frames are shown in the statistic Table S1.
Data exclusions	No data were excluded for this study.
Replication	Data were replicated using technical and independent replicates. See figure legends for specific details.
Randomization	No data required randomization.
Blinding	No data required blinding.

Reporting for specific materials, systems and methods

We require information from authors about some types of materials, experimental systems and methods used in many studies. Here, indicate whether each material, system or method listed is relevant to your study. If you are not sure if a list item applies to your research, read the appropriate section before selecting a response.

Materials & experimental systems

n/a	Involved in the study
☒	☐ Antibodies
☐	☒ Eukaryotic cell lines
☒	☐ Palaeontology
☒	☐ Animals and other organisms
☒	☐ Human research participants
☒	☐ Clinical data

Methods

n/a	Involved in the study
☒	☐ ChIP-seq
☒	☐ Flow cytometry
☒	☐ MRI-based neuroimaging

Eukaryotic cell lines

Policy information about cell lines

Cell line source(s)	Source for HEK293T cell line is ATCC. HTLA cell lines are derived from Dr. Bryan L. Roth's lab. Spodoptera frugiperda (Sf9) cells are from Expression Systems.
Authentication	All cells used in this study are commercial and were obtained from vendors as indicated in the manuscript. HEK293T were certified by ATCC. HTLA cell lines were derived from Dr. Bryan L. Roth's lab.
Mycoplasma contamination	Cell lines were certified as mycoplasma-free as they were obtained from the source companies and labs. See Methods for sources.
Commonly misidentified lines<br>(See ICLAC register)	No commonly misidentified cell lines were used.
